# An Algebro-Topological Description of Protein Domain Structure

**DOI:** 10.1371/journal.pone.0019670

**Published:** 2011-05-24

**Authors:** Robert Clark Penner, Michael Knudsen, Carsten Wiuf, Jørgen Ellegaard Andersen

**Affiliations:** 1 Center for the Topology and Quantization of Moduli Spaces, Department of Mathematical Sciences, Aarhus University, Aarhus, Denmark; 2 Departments of Mathematics and Physics/Astronomy, University of Southern California, Los Angeles, California, United States of America; 3 Bioinformatics Research Centre, Aarhus University, Aarhus, Denmark; 4 Centre for Membrane Pumps in Cells and Disease, Aarhus University, Aarhus, Denmark; University of California, Berkeley, United States of America

## Abstract

The space of possible protein structures appears vast and continuous, and the relationship between primary, secondary and tertiary structure levels is complex. Protein structure comparison and classification is therefore a difficult but important task since structure is a determinant for molecular interaction and function. We introduce a novel mathematical abstraction based on geometric topology to describe protein domain structure. Using the locations of the backbone atoms and the hydrogen bonds, we build a combinatorial object – a so-called *fatgraph*. The description is discrete yet gives rise to a 2-dimensional mathematical surface. Thus, each protein domain corresponds to a particular mathematical surface with characteristic *topological invariants*, such as the genus (number of holes) and the number of boundary components. Both invariants are global fatgraph features reflecting the interconnectivity of the domain by hydrogen bonds. We introduce the notion of robust variables, that is variables that are robust towards minor changes in the structure/fatgraph, and show that the genus and the number of boundary components are robust. Further, we invesigate the distribution of different fatgraph variables and show how only four variables are capable of distinguishing different folds. We use local (secondary) and global (tertiary) fatgraph features to describe domain structures and illustrate that they are useful for classification of domains in CATH. In addition, we combine our method with two other methods thereby using primary, secondary, and tertiary structure information, and show that we can identify a large percentage of new and unclassified structures in CATH.

## Introduction

Protein domains are protein subsequences that may fold and function independently of the rest of the protein [1], [2]. Experimentally determined protein structures deposited in PDB [3] have been classified according to their fold and function in hierarchical databases of which CATH [4] and SCOP [5] are the most widely used. These databases involve manual steps, assisted by computational methods, for fold characterization and classification [4]–[6]. The database DALI [7], [8], on the other hand, uses a fully automated procedure to classify domains non-hierarchically based on structural similarities only. Other methods have been proposed to reduce the description of a domain fold to a vector of numerical attributes that are characteristic for the fold [9], [10]; recent methods are, for instance, based on geometric characteristics [11]–[14], secondary structure information [15]–[18], sequence information [19], and physical properties derived from the primary sequence [20]. These methods might be useful, not only for classification, but also for annotation and understanding features of protein folding.

Using techniques from geometric topology, we propose a novel mathematical abstraction for studying protein domain structures [21]. In particular, we conceive the structure as a fatgraph [21], [22], which is a graph in the ordinary sense extended in a particular way to be explained below. Fatgraphs have been used for studying various problems in mathematical physics; here we investigate their use for studying complex molecular structures.

**Figure 1:**
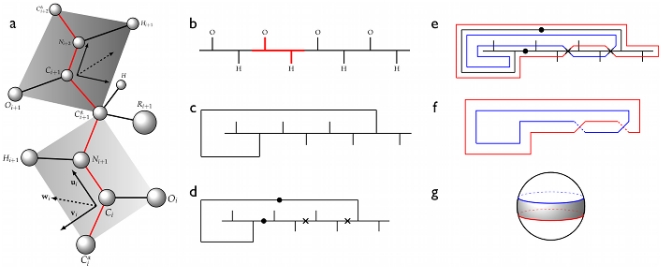
The fatgraph construction. (a) Two neighbouring peptide units of a protein domain labelled as the 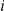-th and 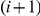-st unit and the vector triple 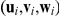 defining the associated coordinate system of unit 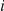. The vector 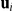 follows the direction of the bond 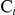
–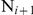, 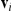 is perpindicular to 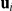 and points towards the same side as 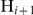, and 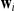 is constructed to form a right-handed coordinate system (b) Concatenated building blocks (one block shown in red), representing a backbone of four peptide units. Vertical stubs correspond to 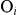 and 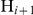. (c) Same as (b) but with one hydrogen bond attached. (d) Hydrogen bonds and alpha carbon linkages are labelled according to the relative orientations of the coordinate frames in (a) such that little change in the orientation results in an untwisted edge (*Materials and Methods*, section 1); 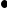 = untwisted and x = twisted. (e) The fattening of the graph is illustrated by colored lines depicting the margins (boundaries) of the strip. The strip is twisted whenever a twisted bond is encountered. (f) The band in (e) with the underlying graph removed. (g) The two adjacent twists cancel out, resulting in band similar to a sphere with two discs removed. It has two boundary components (blue and red). In (c) the hydrogen bond may also be drawn around the right end of the backbone or even cross over or under. The fatgraph is not sensitive to how the hydrogen bonds are drawn and all possibilities yield the same surface.

**Figure 2:**
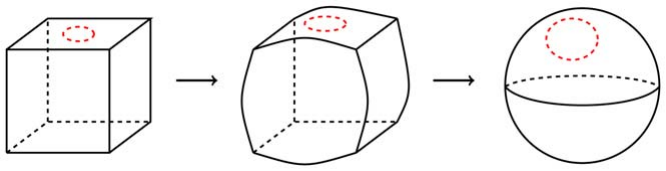
Inflation of a cube with one boundary component constitutes an example of a homeomorphism. The inflation happens without breaking the surface, and only bending and stretching are used.

The construction of a fatgraph corresponding to a protein domain is illustrated in Fig. 1. The peptide unit is the basic unit of description in our model disregarding the amino acid residue. In Fig. 1a, the 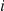-th and 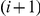-st peptide units of a protein domain are shown. Each peptide unit is a planar region [23] and is represented as a building block with two stubs corresponding to the oxygen and hydrogen atoms (Fig. 1b). The domain backbone is thus depicted as a series of concatenated building blocks. Fig. 1c shows four such building blocks with one hydrogen bond between two peptide units indicated by an edge connecting the H-stub of the first building block with the O-stub of the last. Subsequently, each edge (hydrogen bond and link between building blocks, termed *alpha carbon linkage*) is considered as twisted or not twisted (untwisted) depending on the relative orientations of the peptide units in physical space (Fig. 1d; *Materials and Methods*, section 1). Finally, each edge is widened to become a strip (Fig. 1e), hence the term *fat*graph. The strip is twisted whenever a twisted bond is encountered, similar to how a piece of paper is twisted when forming a Möbius band. In Fig. 1f, the surface is shown without the underlying fatgraph. The two twists in Fig. 1f cancel each other, and the resulting strip is equivalent, *homeomorphic* in the parlance of topology, to a sphere with the regions around the North and South poles removed (Fig. 1g and *Materials and Methods*, section 2). The concept of a homeomorphism is exemplified in Fig. 2, where a cube is continuously transformed into a sphere. This also illustrates why geometric topology is often referred to as *rubber-sheet geometry*.

In general, the surface corresponding to a fatgraph is homeomorphic to a particular 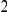-dimensional surface, implying that a fatgraph can be studied by algebraic topological methods and categorized using concepts going back to the work of Leonhard Euler in the 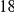
th century [21], [22], [24]. For example, when allowing self-intersections during deformation as well as insertions and deletions of full twists, the resulting class of surfaces is uniquely determined by its genus 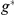, number of boundary components 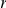, and whether it is *orientable* or not [24]. A surface is called orientable if it is possible to define a consistent orientation (e.g. defined by the right-hand rule) on the entire surface. The strip in Fig. 1 is orientable, whereas the Möbius band is not: One may start a walk from any point and come back again upside-down. The variables 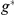 and 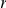 are examples of *topological invariants* which are quantities that do not change when the surface is bent or stretched (i.e. changed under homeomorphic transformations). The Euler characteristic, defined for any surface as 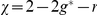, is another invariant summarizing the overall shape of a surface in a single number. In the special case of surfaces arising from fatgraphs of proteins, the Euler characteristic can be computed directly from the fatgraph. In fact, one may show that 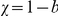, where 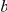 is the number of hydrogen bonds [21]. As demonstrated in Fig. 1e, the number of boundary components is easily counted, whereas the modified genus is less transparent. However, by using the two alternative descriptions of the Euler characteristic, we may express 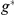 in terms of simpler quantities, 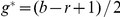. Thus, 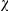 has direct biologial interpretation (in terms of number of hydrogen bonds) whereas 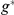 and 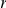 are quantities derived through the fatgraph abstraction.

The surface in Fig. 1 is a sphere (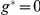) with two boundary components (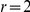), one for each of the removed discs (that is the North and South poles), and the structure has only one hydrogen bond. In particular, the alternative expression 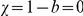 agrees with 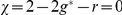. The fatgraph abstraction thus opens an entirely new perspective on protein structure by replacing complex structures by much simpler constructs.

**Figure 3:**
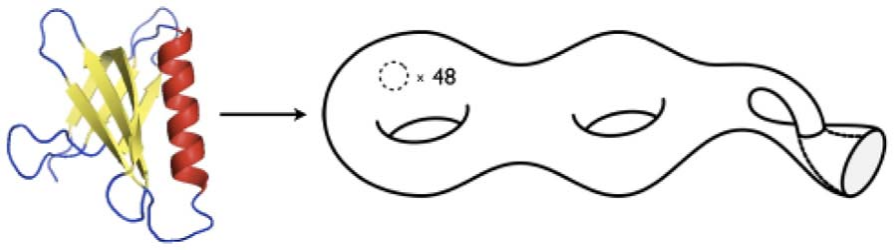
The protein domain 1ptoF00 is an alpha-beta domain classified as *OB fold* (Dihydrolipoamide Acetyltransferase, E2P; 2.40.50). The corresponding surface is not orientable and has genus 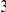 and 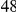 boundary components. The surface is homeomorphic to two tori connected to a Klein bottle with 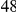 discs removed. The surface is difficult to visualize in 3D; in the figure the handle crosses through the bottle with no physical contact.

An example of a surface corresponding to a domain fatgraph is shown in Fig. 3. The CATH protein domain 1ptoF00 is a mixed alpha-beta domain classified as *OB fold* and has 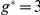 and 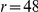. The corresponding surface is non-orientable and thus has only one side (up and down are the same), just like a Möbius band. Furthermore, the surface has 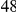 discs cut out, each giving rise to a boundary component.

We demonstrate that the *global* variables 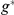 and 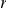 capture structural differences in domains. We show this by example and also by analysis of the distributions of 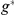 and 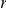 values over domains. Further, we illustrate how 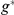, 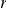 and other fatgraph variables can be utilized for classification. In addition to the global variables, the fatgraph abstraction allows us to introduce a simple local or secondary structure annotation, namely the backbone as a sequence of twisted and untwisted edges. Using machine learning techniques, the usefulness of the fatgraph abstraction is illustrated by classification of domains in the CATH database. We compare to an alternative geometric approach [11] and to an approach based on sequence information only. We show that combining information from all three makes a very strong classifier. Further, we investigate the causes of false predictions and show that our methods are able to detect domains in v3.3.0 with classifications that are non-existing in the previous version, v3.2.0. The classification scheme devises a method for flagging domains as possible new or problematic folds.

## Results

### Robust variables

**Figure 4:**
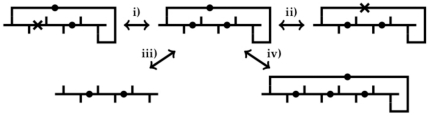
The four basic modifications of fatgraphs. Variables defined on a fatgraph that change at most linearly with the number of basic modifications are called robust. i) Change the color of a bond between peptide units, ii) Change the color of a hydrogen bond, iii) Add or remove an untwisted hydrogen bond, and iv) Replace a fatgraph building block by two building blocks connected by an untwisted alpha carbon linkage, where any edges corresponding to hydrogen bonds incident on the original building block are connected to the replacement building block that occurs first along the backbone from N to C termini, and the reverse of this operation. Any fatgraph can be created from an arbitrary starting fatgraph by repeated application of i)–iv).

For each domain we compute the corresponding fatgraph and calculate four robust variables derived from it (*Materials and Methods*, section 3 and Fig. 4). Robust variables are defined such that they are relatively insensitive to noisy and imprecise experimental data; that is, noise in data that may result in errors in the fatgraph.

We represent a domain by four robust variables, among these the genus 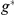 and the number of boundary components 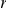 of the corresponding surface. These variables are global in the sense that they cannot be related to any particular region of the domain. Furthermore, we consider the domain length 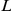, measured as the number of amino acid residues, and the number of twisted alpha carbon linkages 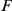 that measures how often the backbone twists in the orientation of the planar peptide units (Fig. 1d). Recall that insertions and deletions of full twists are not captured in 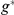 and 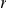, but this is compensated for in 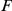 (Fig. 1f).

The CATH database classifies domains in a hierarchical scheme with four main levels (listed from the top and down) called class (C), architecture (A), topology (T), and homologous superfamily (H), hence the name CATH [4], [25]. At the C-level domains are grouped according to their secondary structure content into four categories with the three main ones being *mainly alpha*, *mainly beta*, and *mixed alpha-beta*. The last category contains domains with only very few secondary structures. The A-level groups domains according to the general orientations of their secondary structures, and at the T-level the connectivity (the order) of the secondary structures is taken into account. The grouping of domains at the H-level is based on a combination of both sequence similarity and a measure of structural similarity. Below the four main lavels, CATH has an additional five layers called S, O, L, I, and D. The first four group domains according to increasing sequence overlap and similarity, and the D-level assigns a unique identifier to every domain thus ensuring that no two domains have the exact same CATHSOLID classification.

**Figure 5:**
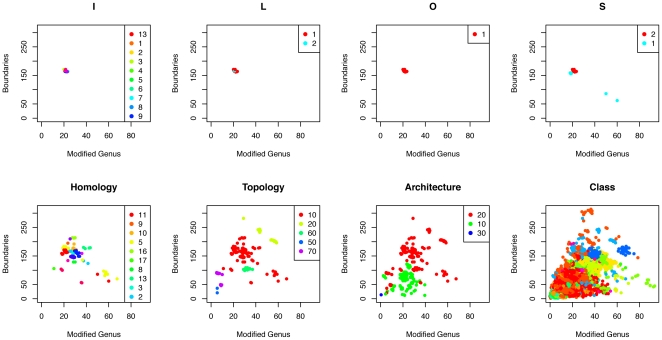
The domain 1o88A00 is classified as Pectate Lyase C-like (2.160.20) with complete CATHSOLID classification 2.160.20.10.11.2.1.1.1. The Class plot shows 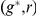 for all domains with 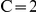 (colored according to A-level), and the Architecture plot shows 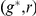 for all domains with 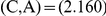 (colored according to the three T-levels). This continues all the ways down to the last plot where 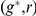 are shown for for 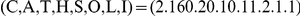.

**Figure 6:**
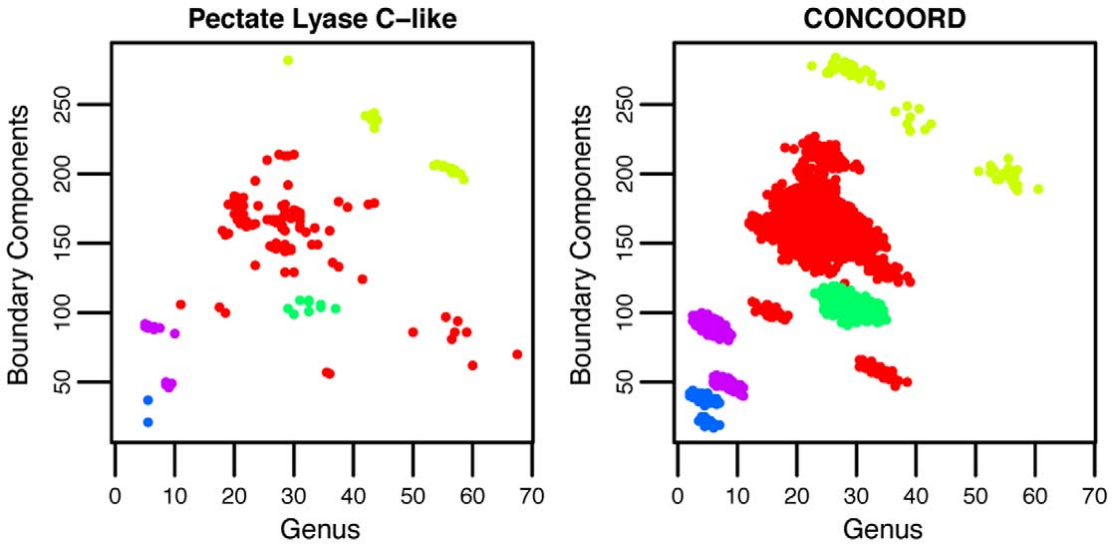
Robustness of topological invariants to noise. Left: Scatter plots of 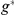 and 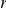 for domains in CATH topology *Pectate Lyase C-like* (2.160.20). The five H-levels are indicated by different colors (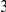 to 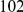 domains in size), and a clear separation of H-levels is observed. Right: 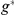 and 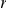 calculated from CONCOORD modified structures. Even with noise, separation at H-levels is still clearly visible. Note that in the bottom right corner in the left figure there are eight red dots without counterparts in the right figure. The number of hydrogen bonds typically increases when a domain is modified using CONCOORD, but the eight domains corresponding to the missing dots each showed a decrease in the number of hydrogen bonds (Fig S1).

Fig. 5 shows an example of how 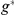 and 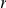 separate domains at different CATHSOLID levels. It transpires that the best separation is obtained at T-, H-, and S-levels. The grouping at the A-level is often very broad, and an architecture may comprise domains of very different sizes. Furthermore, since the order of the secondary structure elements is not taken into account at the A-level, a single architecture may contain domains with very different connectivities [4], [25]. This is likely the explanation for the lack of separation of A-levels observed in Fig. 5. On the other hand, because the fatgraph approach is based on structural features, we do not expect to see a clear separation at the SOLID levels, since these are defined in terms of sequence overlap and similarity. Fig. 6 shows that 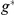 and 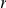 separate the H-level families in the CATH topology *Pectate Lyase C-like* (CATH classification 2.160.20) with one family (red in Fig. 6) being larger than the others. To test the empirical robustness of the variables, we generated 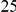 modified structures for each domain using the CONCOORD algorithm [26] and calculated 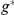 and 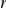 from the resulting structures (Fig. 6 and *Materials and Methods*, section 4). The figure indicates that even after modifications, the variables are able to separate domains at the H-level. Furthermore, for individual domains, the variables did not in general deviate significantly from the original values (illustrated in Fig. S2).

### Distribution of fatgraph variables

**Figure 7:**
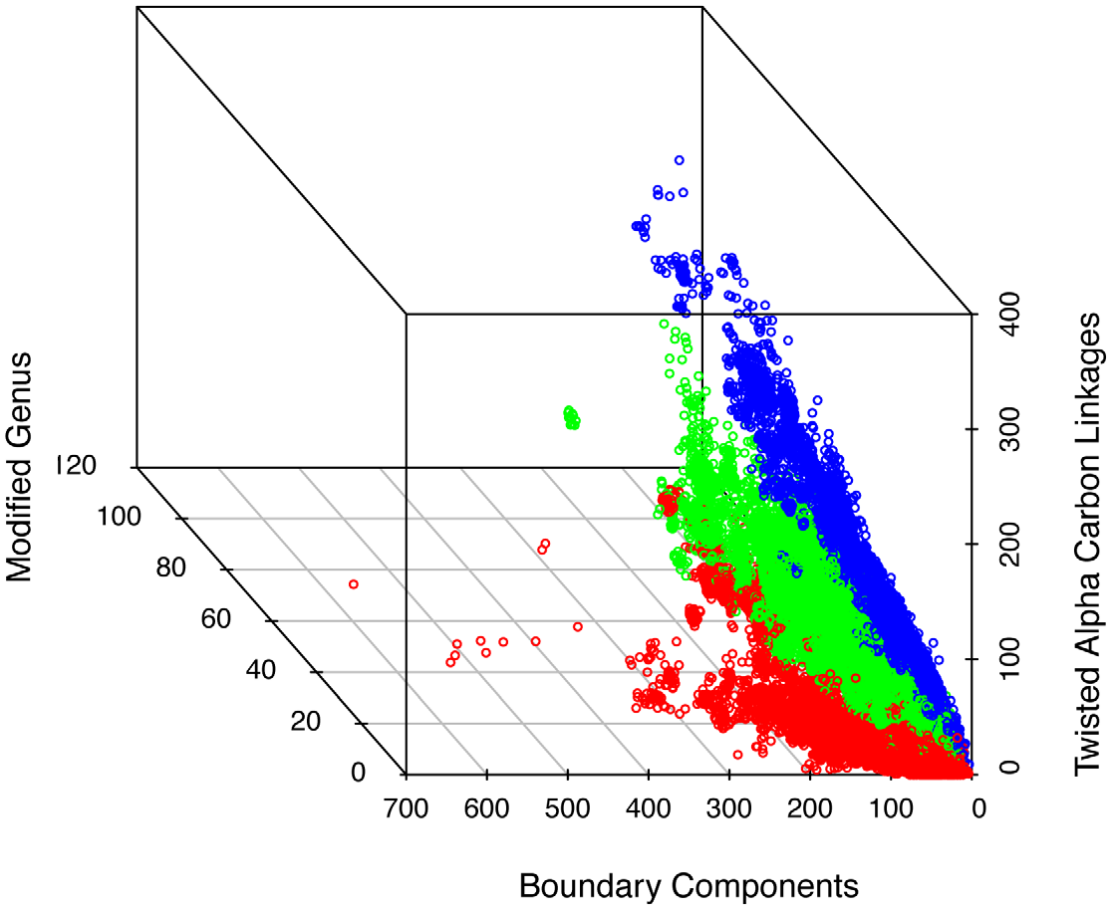
Scatter plot of 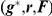 for all domains in the three main classes mainly alpha (red), mainly beta (blue), and mixed alpha-beta (green). Visually, the mainly alpha and mainly beta domains are separated with the mixed alpha-beta domains residing in between.

Fig. 7 shows a scatter plot of 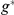, 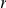, and 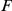 for the three main classes (C-level) in v.3.3.0. Generally, the mainly alpha domains have lower 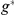 and higher 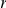 than the mainly beta domains with the mixed alpha-beta domains falling in between. For example, mainly alphas have many domains with 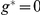 corresponding to a sphere with 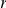 discs cut out. For small values of 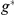 and 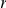, almost all combinations are found, but for higher values, only a small fraction of all possible combinations are observed. More details are shown in Figs. S3–S4, with pairwise scatterplots of the variables 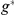, 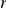, 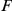, 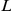, and the Euler characteristic 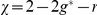. Empirically, fairly sharp boundaries appear for possible values, and longer domains tend to have higher values of both 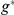, 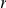, and 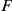 than shorter domains. A total of 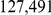 domains are non-orientable, and only 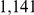 domains are orientable. We expect this because a single twisted hydrogen bond may introduce a Möbius band and potentially alter the orientability of the corresponding surface: For example, in Fig. 1 the two adjacent twists cancel out, and the surface becomes orientable, but removing one of the existing twist or adding an extra twist along the backbone results in a Möbius band. Similarly, moving the right-most end of the hydrogen bond one stub to the left, thus separating the two twists, yields two Möbius bands which do not cancel out.

**Figure 8:**
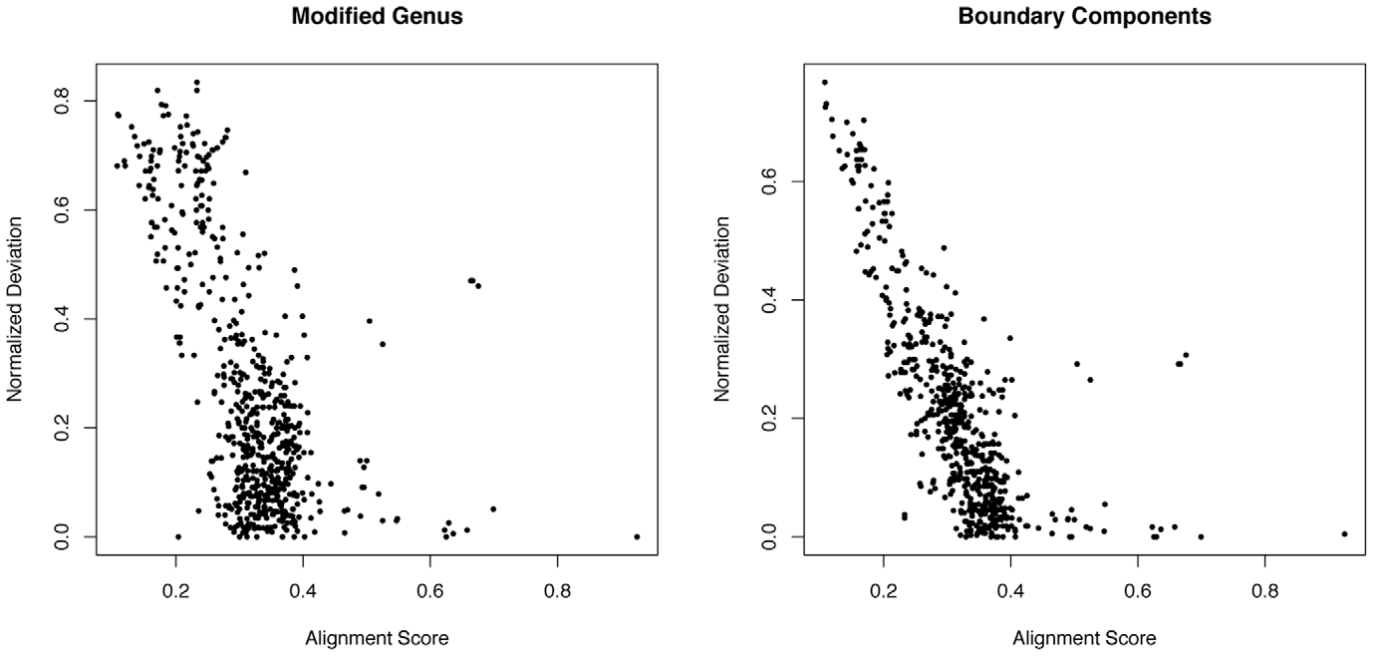
Alignment scores versus differences in 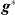 and 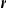 for all pairs of S95-domains in the Pectate Lyase C-like topology (2.160.20). We use the normalized difference 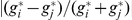 between modified genera (and similarly for boundary components) to take discrepancies in domain length into account. A high alignment score indicates high sequence similarity and the plot illustrates that similarity is at the primary and tertiary levels are correlated.

Structural divergence may be caused by only modest modifications at the amino acid sequence level, and we compared how differences in sequences are reflected in the topological invariants. Fig. 8 shows scatter plots of normalized alignment scores (*Materials and Methods*, section 7) versus normalized differences in 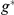 and 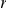, respectively, for all pairs of S95-domains in the topology Pectate Lyase C-like (2.60.120). In general, low sequence similarity implies relatively large differences in 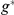 and 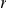 with only a few outliers. For example, three domains have sequences very similar to that of 2iq7A00 (alignment score 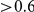), but still the normalized differences in 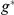 (resp. 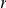) are almost 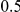 (resp. 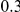). This may be explained by a lower number of hydrogen bonds in 2iq7A00 compared with the three other domains – a feature captured by the topological invariants but not by sequences alone.

To further assess the ability of the four fatgraph variables to distinguish different folds, we performed pairwise Wilcoxon tests comparing the distributions of each variable using the 1,161 H-level families in v3.3.0 containing ten or more domains (in total 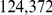 domains or 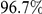 of all domains). The results are summarized in Fig. S5, and the plot indicates that in general the four variables are sufficient to distinguish most H-levels. In fact, at significance level 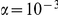, almost all pairs of H-levels (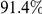) are distinguishable by at least three of the four variables.

### Secondary structure elements

**Figure 9:**
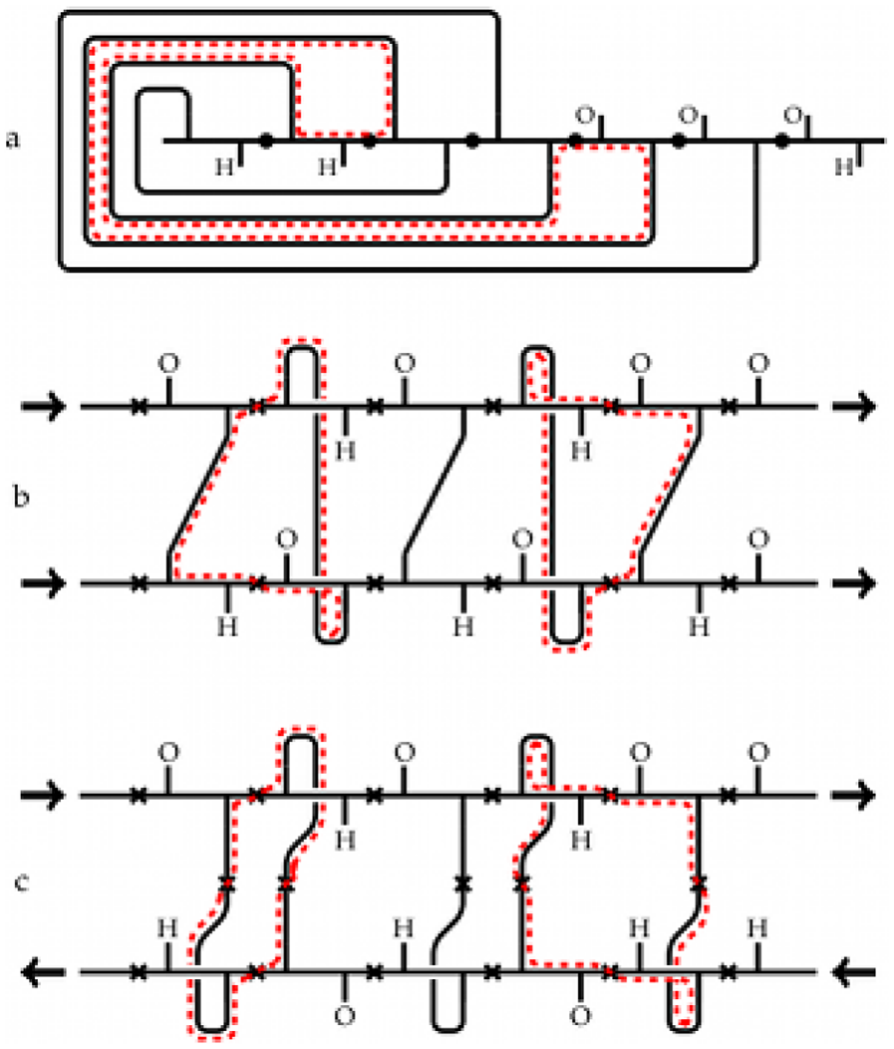
Fatgraphs corresponding to the common secondary structures. (a) alpha helix, (b) parallel beta sheet, and (c) anti-parallel beta sheet. Each boundary component (dashed red line) passes through four peptide units; the alpha helix is local in that it connects only closely situated peptide units whereas the beta sheet also connects peptide units potentially far away from each other. The number of components depends on the length of the structure. The less frequently occurring 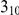-helices and 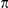-helices give rise to similar pictures as (a), the only difference is that hydrogen bonds connect stubs three and five peptide units apart instead of four. The backbone of an 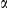-helix is a string of untwisted alpha carbon linkages, whereas for the 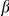-sheets these are twisted.

The secondary structure is a particularly rigid part of a protein structure, and this is reflected in the corresponding fatgraph. Fig. 9 depicts idealized fatgraphs arising from the three most common secondary structure motifs: (a) alpha helices, (b) parallel beta sheets, and (c) anti-parallel beta sheets with typical boundary components indicated by dashed red lines. All boundary components pass through exactly four different peptide units, and the backbone of an alpha helix consists of untwisted edges, whereas the backbone of a beta sheet consists of twisted edges.

A domain consisting of one long alpha helix has genus zero, and the number of boundary components is proportional to the length of the domain. Likewise, a beta sheet contributes to the number of boundary components proportionally to the sheet size and only marginally to the genus. The apparently abstract topological quantities thus exhibit direct relationships to the secondary structures of the domains (Fig. S6). This observation agrees with the empirical result above that the main CATH classes show differences in the distribution of 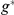 and 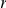.

### Non-additivity of fatgraph variables

The topological quantities corresponding to an entire domain cannot be obtained directly by adding quantities from individual secondary structure components alone; most domains have stabilizing hydrogen bonds between secondary structure elements, and these contribute in a non-linear fashion to the fatgraph. This non-additivity is e.g. reflected in the mainly alpha class (Fig. S6), where the genus increases with increasing number of alpha helices (despite each has 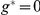) because the helices are stabilized by bonds between them.

**Figure 10:**
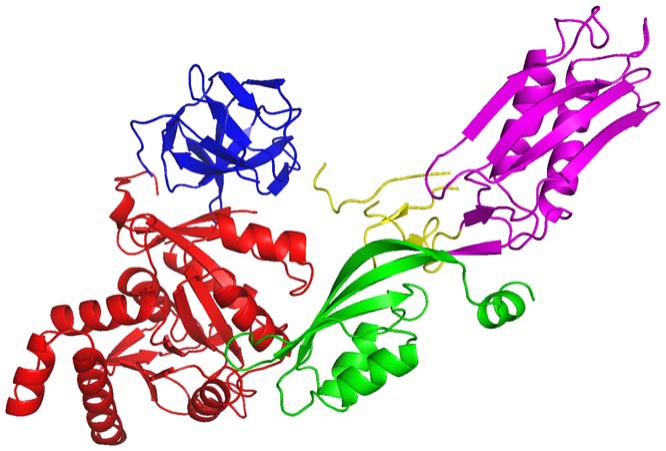
The protein 1DAR comprises five CATH domains with individual genera 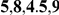, and 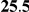, and 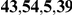, and 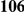 boundary components. The entire protein considered as one contiguous structure has 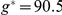 and 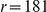, but the sums of the individual 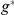 and 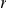 are 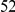 and 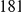. The robust variables 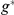 and 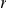 are thus not additive. The figure is made using PyMOL (www.pymol.org).

The lack of additivity is perhaps even clearer when considering entire proteins comprising multiple domains. As a concrete example, consider the protein 1DAR (Fig. 10) with five CATH domains. Considered as one contiguous structure, 1DAR has 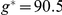 and 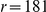, but the genera and boundary components corresponding to the individual domains add up to 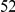 and 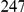, respectively. Examples where the genus (resp. the number of boundary components) of the entire protein is smaller (resp. larger) than the sum of those corresponding to the individual domains also exist.

Despite the relatively high deviation of 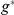 and 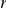 from the sums obtained from the individual constituents of a structure, the Euler characteristic 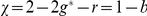 is generally more consistent. In the example 1DAR, the sum of the Euler characteristics is 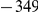 compared to 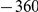 for the entire protein. If there are 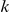 domains, then
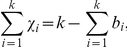



where index 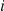 refers to the 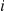
th domain, 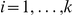. That is, the difference between 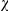 of the entire protein and the sum is 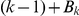, where 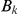 denotes the number of bonds *between* domains. Since, in general there are fewer hydrogen bonds between domains than within, the sum is close to 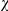 of the whole protein.

### Classification using robust variables

We attempted to reproduce the CATH classification using only the four robust variables, 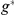, 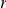, 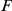, and 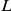. We applied different classification techniques to the data and found that the method Random Forests [27] generally performed well.

**Figure 11:**
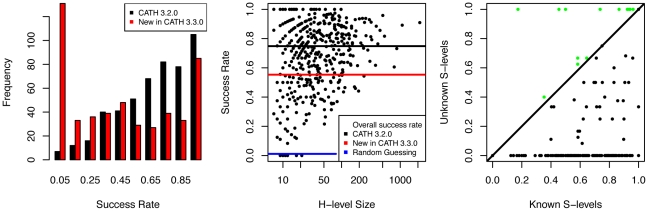
Classification at H-level using 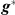, 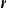, 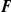, and 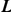. Left: Distributions of success rates for the 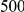 largest H-levels in the CATH 3.2.0 test set (black) and the new domains in CATH 3.3.0 (red). Middle: Success rates for the CATH 3.2.0 training set plotted against H-level sizes. Average success rates are indicated by lines. Right: In the CATH 3.2.0 test set, 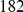 H-levels contain domains with S-levels not present in the training set. In all but 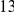 cases (green), the classifier performs better on the domains with known S-level.

**Figure 12:**
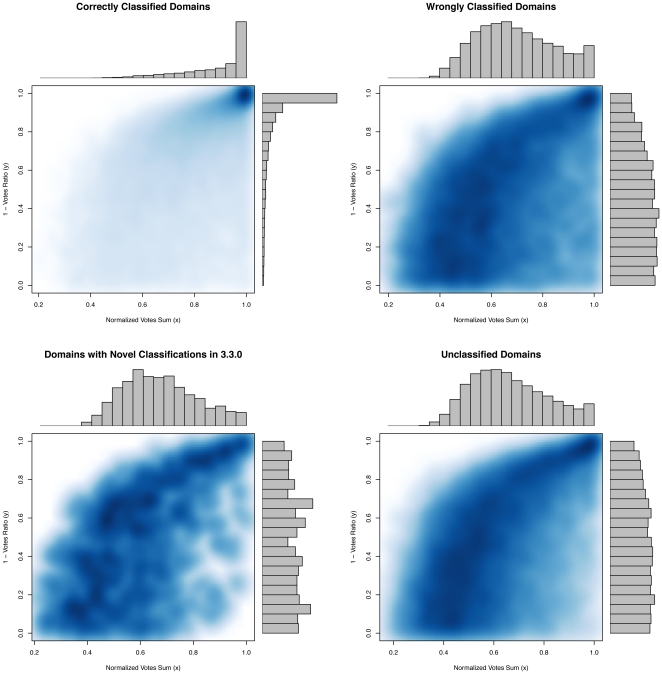
The classifier consists of a collection (forest) of classification trees. For each domain in the test set, each classification tree votes, and a consensus is reached. We used 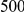 trees, and for each domain we calculated the percentages, 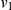 and 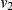, of the most frequent and the second most frequent votes occuring. The top plots show the distributions of 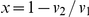 and 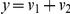 for the correctly and wrongly classified domains at the H-level in v3.2.0. If 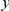 is large, the majority of votes are cast for the top two candidates, and if furthermore 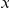 is large, many more votes have been cast for the winner compared to the runner-up. Therefore, if both 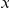 and 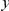 are large, this indicates that the classifier makes a confident prediction. The distributions of 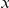 and 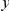 for correctly predicted domains show that the confidence in correct predictions is generally high. On the other hand, confidence in wrong predictions is much more uniform. The bottom plots show the distributions corresponding to the 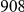 newly added domains in v3.3.0 with non-existent classification in v3.2.0 and the 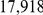 domains for which CATH has not yet provided a classification. Both show the same kind of uncertainty as observed for the wrongly classified domains.

In v3.2.0 (SAll, see *Materials and Methods*, sections 5), we selected the 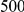 largest H-levels (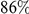 of all domains) and randomly sampled 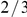 of the domains for training, while keeping 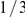 for testing (*Materials and Methods*, sections 5 and 6). In addition, we tested the classifier on the new domains in v3.3.0 that are not already in v3.2.0. Fig. 11 shows the results. We assigned 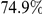 of the domains in v3.2.0 into their correct H-level, whereas 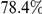, 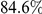 and 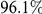 are correctly assigned at the T-, A-, and C-level, respectively. For the new domains in v3.3.0, the percentages are smaller: 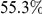 (H), 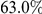 (T), 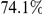 (A), and 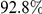 (C). When the classifier makes a correct prediction, it does so with high confidence whereas it is less certain when making a false prediction, and a similar lack of confidence is observed when the classifier is applied to domains which have not been assigned a classification by CATH or domains with H-levels that are new in v3.3.0 (Fig. 12).

Performances for other selections of variables at the H-level are shown in Fig. S7. We found that the domain length is an important variable for correct prediction (an observation also made in [11]). We also varied the energy cut-off used to infer hydrogen bonds (*Materials and Methods*, section 1). The resulting values of the robust variables are strongly correlated with those calculated using the default cut-off, and the classification results did not change significantly (Fig. S7).

Some of the largest families show a remarkable homogeneity in 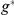
- and 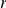-values across domains (Fig. S8), which to some extent stands in contrast to reports indicating structural diversity within H-levels based on RMSD measures [28]. Our approach may therefore provide an important complement to existing classification schemes.

The performance is generally lower on the new domains in v3.3.0 than on the domains in v3.2.0. The lower performance could in principle be due to skewness in family sizes in the two data sets, but this is not observed (Fig. S9). To further explore this discrepancy, we used the S-level immediately below the H-level as a proxy for the complexity of the H-levels. Fig. 11 shows that the classifier performs much better on domains with known S-level (i.e., S-levels that are associated with domains in the H-level training set) than on domains with unknown S-level (i.e., S-levels not found for any domain in the training set). Furthermore, 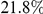 of the new domains have unknown S-levels while this was only the case for 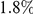 of the domains in the v3.2.0 testing set. In the training set, this must be due to sampling whereas in the new set, the difference is mainly caused by genuinely new S-levels introduced in v3.3.0. This finding indicates that the known S-levels and the S-levels new in v3.3.0, despite being defined based on sequence similarity, also differ in their fatgraph characteristics.

### Classification using flip sequences

The domain example in Fig. 1d comprises three alpha carbon linkages, one untwisted and two twisted. Reading from left to right, the conformations may be represented as a string UTT with U (T) meaning untwisted (twisted). In this way, each CATH domain has an associated sequence of these letters of length one less than the number of peptide units, and we refer to this sequence as the *flip sequence*. Alpha helices and beta sheets have particularly simple flip sequences, namely, UUU… and TTT…, respectively (Fig. 9).

The alignment score computed from an alignment of two flip sequences gives a measure of similarity between the corresponding domains, and we applied this score to build an alternative CATH classifier using flip sequences (secondary level) rather than robust variables (tertiary level). To do so, we randomly selected 2/3 of all domains in v3.2.0 (S95 or SAll; *Materials and Methods*, section 5) for training, keeping 1/3 for testing. In addition, the domains that are new in v3.3.0 or unclassified in v3.3.0 were also kept for testing.

For all domain pairs 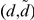 in the training set, we calculated the pairwise, normalized alignment score 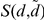 (*Materials and Methods*, section 7). Subsequently, we defined the similarity between a domain 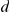 and an H-level 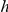 (similarly for C-, A-, and T-levels) as



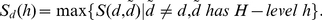
(1)


**Figure 13:**
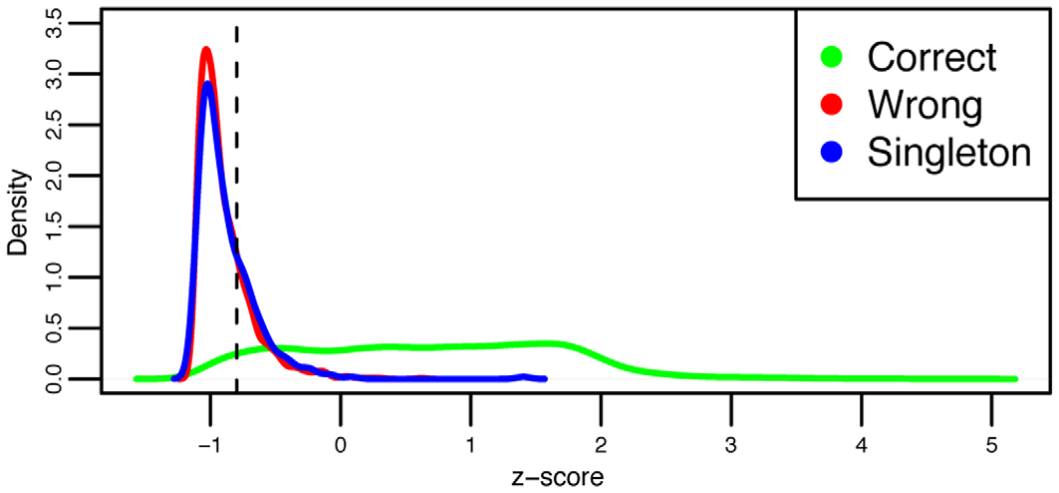
The plot shows the 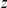-score l for each domain in the CATH 3.2.0 training set (S95) for H-level, based on flip sequences. The vertical dashed black line indicates the 95% sensitivity decision threshold; if the 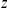-score of a domain is above the line it is assigned the classification of the nearest H-level, otherwise it is left unassigned. H-levels with only one domain in the test set cannot be classified in this way. Green = Closest H-level is correct, Red = Closest H-level is wrong, Blue = Unclassifiable.

For each 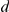, we identified the two H-levels with the highest scores (Fig. 13). In the figure a green (red) dot indicates that the H-level with the highest score is the same (not the same) as that of 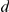. The relationship between the scores is clearly indicative of the H-level of 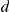. The scores for the two nearest H-levels were combined into one variable (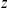-score) and a test was designed to facilitate comparison between methods (*Materials and Methods*, section 7). The same procedure was adapted to amino acid sequences (primary structure). Additionally, we compared to a geometric method for classification that uses tertiary structure information [11] in the following way: A domain is represented as a 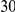-component vector comprising the number of residues and 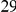 quantities derived from a geometric description of the backbone curve. The domain backbone is viewed as a piecewise linear curve in three dimensions with each piece corresponding to a bond, and the average number of crossings when the curve is viewed from all possible angles is computed. This *average crossing number* is one of the 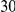 variables used in the classification, and except the number of residues, the remaining 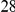 variables are all generalizations (known as *Gauss integrals*) of the average crossing number.

Overall the results are comparable (Table 1 for S95); further results for S95 and SAll are shown in Table S1 with amino acid sequences generally performing better than Gauss integrals and flip sequences. All methods show a decrease in sensitivity and specificity for the domains that are new in v3.3.0 (Table S1), which is similar to that observed for the robust variables though less pronounced and attributed to differences between the old and new domains, such as S-levels, indicating that the new domains are evolutionary more diverse than the old ones.

**Table 1:** Comparison of methods.

Method	C	A	T	H
Gauss Integrals	95.3/95.3/42.0/NA	89.0/95.5/36.1/NA	85.2/95.0/43.5/47.1	80.8/95.7/30.9/34.3
Flip Sequences	94.0/95.3/33.0/NA	82.4/95.1/15.7/NA	75.4/94.6/21.0/22.6	73.0/94.9/19.0/24.3
Amino Acid Sequences	86.2/95.4/17.4/NA	79.8/94.9/22.1/NA	79.2/95.3/28.9/31.3	80.6/95.1/38.8/35.0
Combined	95.8/95.2/69.6/NA	90.3/95.3/62.7/NA	87.9/95.4/69.6/67.8	87.2/95.4/70.4/70.7

Each cell contains performance, sensitivity, specificity, and the number of *unknown* domains flagged correctly (in percent) in that order on the CATH v3.2.0 test set at each CATH level for all three methods. Decision thresholds were calibrated on the CATH 3.2.0 training set to achieve 95% sensitivity. Levels comprising only a single domain in CATH 3.2.0 are never included in the training set and thus account for the numbers in the unknown column; for C- and A-levels, there are none of these.

**Figure 14:**
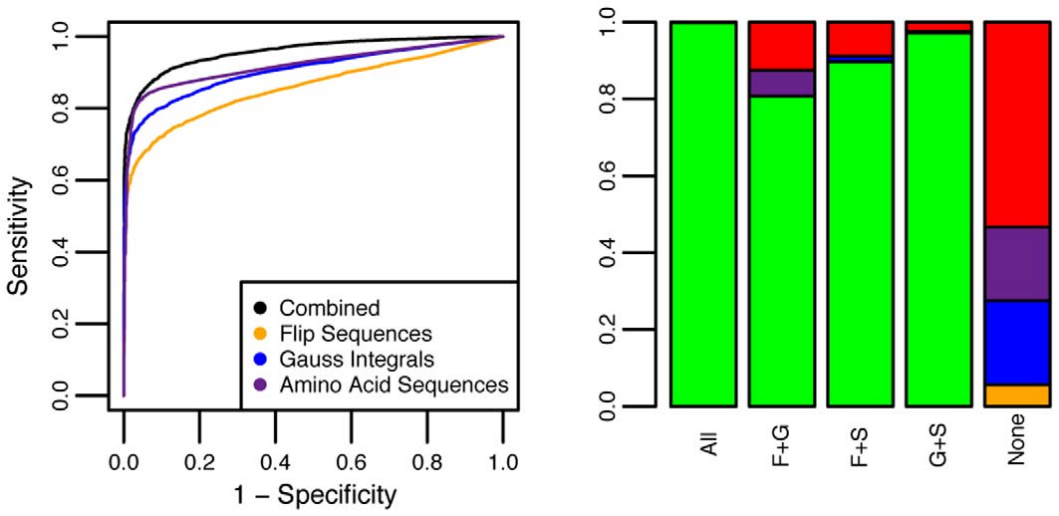
Analysis of sensitivity and specificity. Left: ROC curves for S95 for the three tests individually and the combined test. Combining information from all three clearly improves sensitivity and specificity considerably. Right: The columns show how often agreeing methods (S = amino acids, F = flips, G = Gauss) are correct (green), and how often the non-agreeing method is correct; S (purple), G (blue), F (orange). Red indicates the number of times none of them are correct. The numbers in each column are 6057 (F+G+S), 476 (F+G), 348 (F+S), 697 (G+S), and 2084 (None).

We combined all three tests into one to achieve higher performance (*Materials and Methods*, section 7, and Fig. 14). For S95, the AUC (area under curve) increases to 96% for the combined test from 86% (flips), 90% (Gauss), and 91% (amino acids) for the individual methods. In particular, for a sensitivity of 95%, the specificity is at least 50% higher for the combined test than on any of the individual tests individually. Likewise, the combined test is able to identify 78.4% of all domains with unknown H-level in v3.2.0, and 46.6% of all domains that are unclassified in v3.3.0. For SAll, the latter percentages raise to 100% and 92.8%, respectively, making the classifier very capable of detecting domains with structures potentially not in CATH v3.3.0.

## Discussion

We discuss a new representation of protein structure and show how local and global variables vary across domains and structural classification. The description makes use of concepts from geometric topology and represents a domain structure by a fatgraph that in turn can be interpreted as a 2-dimensional surface. The structure of the fatgraph relies on atomic coordinates of the protein and its hydrogen bonds but not on primary sequence information.

The representation provides a complementary and alternative view to structures as purely 3D geometric objects [7], [8], [11]–[14] and shows several strengths. The domain structure is conceptualized in terms of entities that are amenable to further manipulation and characterization; for example, we compute the genus and the number of boundary components that are both topological invariants. Even though several unrelated domains may share the same invariants, we are able to classify the majority of the domains correctly. Further, we have introduced the idea of a robust variable, namely, a variable defined on the fatgraph that is robust towards noise and errors in the experimental determination of the structure, and we have formulated robustness in terms of operations on the fatgraph and investigated the robustness empirically.

The invariants 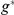 and 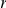 of a domain cannot in general be computed from the secondary structures alone. Due to stabilizing bonds connecting secondary structure elements, 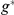 and 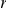 may differ significantly from what is obtained by summing the genera and the number of boundary components of individual secondary structure elements. The invariants thus capture tertiary structure information.

We showed that using secondary structure information we could classify a large percentage of domains in S95 (and SAll) correctly. However, correct (and better) classification could also be achieved using other methods using primary or tertiary structure information. We combined all three methods and achieved a method with higher performance as well as sensitivity and specificity. The combined method is also able to identify the majority of unknown or unclassified domains.

We used the CATH database as a gold standard, but using appropriate clustering algorithms it would be possible to make a *de novo* classification and compare this to existing classification. Given the observation that classification based on primary structures is best at reproducing the CATH database, it is conceivable that a *de novo* classification based on structural properties alone would lead to a different hierarchy. However, a *de novo* classification would require further investigations to e.g. determine the number of classes needed, and this is beyond the scope of this paper. The lack of agreement between the two most widely used databases, CATH and SCOP, certainly indicates that more analyses are needed in order to fully comprehend the universe of domain structures [29].

Automated approaches, including ours, benefit from not relying on human judgment. However, a key difficulty is that structural and evolutionary homology does not always go hand in hand, and different protein families show a wide spectrum in sequence, structural and functional similarities [3], [7], [30]. Currently, the level of classification achieved by manually assisted methods, such as CATH or SCOP, might not be realizable by automated means only, but we believe that advances in mathematical modeling of protein structure will change this in the future and that our model should be a step in this direction. For example, it would be interesting to put our method into a probabilistic setting. In recent papers, it has been shown that probabilistic models might have large potential [31], [32].

Various extension of the fatgraph model are conceivable. For example, one approach could be to include bifurcating hydrogen bonds in the model, as CO and NH groups of the protein backbone engage in two or more hydrogen bonds [23]. However, this phenomenon poses a mathematical question that must be addressed in a biologically meaningful way: The fatgraph model depends on an ordering of the edges around a vertex, and with bifurcating bonds there is no *a priori* way of choosing such orientations. Other extensions could be the inclusion of sulphur bridges, or extending the two types of edges (backbone and hydrogen bonds) to multiple types reflecting more accurately the twisting of the backbone.

## Materials and Methods

### 1. Fatgraph construction

The fatgraph is constructed as explained in the main text, Fig. 1. To each peptide unit we associate a coordinate system (a frame). If the frames of two linked peptide units are similar, the edge connecting them are untwisted and otherwise they are twisted. ‘Similar’ is here measured via a metric on frames, see [21] for details. The procedure is identical to twisting if the sum of scalar products 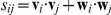 is negative [21], a sum combining the change in the normal vector 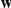 with the change in the orientation of the peptide plane. For example, if the two frames are identical 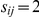 and the edge is not twisted; whereas if frame 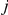 is frame 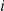 up-side-down, 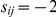 and the edge is twisted.

We infer hydrogen bonds using the DSSP algorithm [33]. The algorithm depends on an energy cut-off, and by default a hydrogen bond is inferred if the electrostatic interaction energy 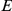 is lower than 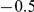 kcal/mol.

### 2. Classification of surfaces and fatgraphs

For integers 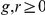, the following families of surfaces are particularly interesting: 1) a sphere with 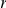 discs cut out, 2) the sum of 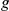 tori with 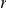 discs cut out, and 3) the sum of 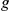 real projective planes with 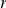 discs cut out. The first two types can be visualized in 3D, whereas the last cannot; 1) is straightforward, and 2) is a series of 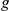 doughnuts glued together with 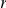 discs removed. Two surfaces are called homeomorphic if one can be transformed into the other by stretching and bending but no tearing. A classical result in algebraic topology [24] states that any closed connected surface is homeomorphic to exactly one of the surfaces above. For example any deformation of a ballon is homeomorphic to a sphere and thus has 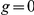. The number 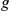 is called the genus, and 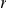 is the number of discs or boundary components of the surface. The surfaces in 1) and 2) are orientable, whereas the surfaces in 3) are not. It follows that a surface is uniquely determined by its 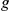, 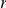, and whether it is orientable. We define the modified genus 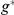 as 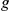 if the surface is orientable and as 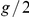 if it is non-orientable [21]. With this definition, the Euler characteristic (a term combining 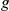 and 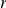) is 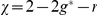 in either case. The number of hydrogen bonds 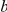 relates to 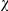 through 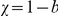.

The invariants, including whether the fatgraph is orientable, can be calculated computationally efficiently and quickly [21]. For example, 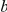 can readily be found from the fatgraph, whereas 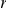 requires more work. For small fatgraphs as the one in Fig. 1, 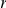 is easily counted, but a more systematic approach must be applied when dealing with larger fatgraphs. This may be accomplished by a purely algebraic approach which is easily implemented in a computer program [21]. Even using a naive and straight-forward implementation, parsing a domain and calculating the corresponding topological invariants takes less than a second on a standard laptop computer. Finally, 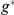 follows from 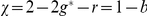. Note that by construction (Fig. 1e), a fatgraph corresponding to a protein always has a least one boundary component, that is 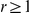. A general surface has 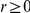 and 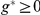.

### 3. Robust variables

A function 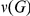 defined on fatgraphs is called 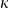-robust for an integer 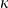, if 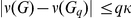 whenever 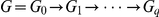 is a sequence of 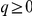 fatgraphs, and 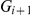 is obtained from 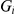 by one of four basic modifications: i) change the label of a bond between peptide units (i.e., remove a twist or introduce a twist); ii) change the label of a hydrogen bond; iii) add or remove an untwisted hydrogen bond; and iv) insert a fatgraph building block 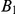 next to an existing building block 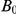 (in the direction from N to C termini), such that the alpha carbon linkage between the two blocks is untwisted, the alpha carbon linkage to the right of 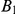 is (un)twisted according to whether the linkage to the right of 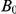 in the original fatgraph is (un)twisted, and 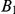 has no hydrogen bonds attached. Or the reverse operation, i.e. removing a block with no hydrogen bonds which has an untwisted linkage to its left neighbour (illustrated in Fig. 4). It can be shown [21] that the functions 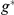, 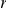, 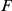, and 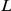 all are 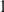-robust. This implies that if 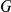 is changed by a sequence of at most 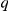 basic modifications, the functions 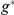, 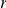, 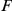 and 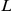 change at most 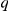.

### 4. CONCOORD modified structures

All modified structures were generated using the CONCOORD algorithm [26] which generates conformations from a known structure based on restrictions on interatomic distances. We used default parameter values.

### 5. CATH domain data

The newest version of CATH (3.3.0) contains 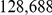 domains whereas the previous version (3.2.0) only comprises 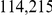 domains. We obtained the classifications of domains in both versions as well as the raw data consisting of chopped PDB-files and corresponding DSSP-files from the CATH homepage (www.cathdb.info). CATH is hierarchical; we focus on the class (C), architecture (A), topology (T) and homologous superfamily (H), and sequence (S) level.

We operate with the following basic data sets: A) CATH 3.2.0, B) the domains in v3.3.0, but not in v3.2.0 (called new in v3.3.0), and C) CATH 3.3.0. In addition, CATH lists 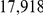 domains that have not yet received CATH classification, either because the domain annotation is questionable or because there is uncertainty about the structural similarity to other domains. We denote this set unclassified in v3.3.0. Among the new in v3.3.0, there are 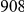 domains with novel H-level, that is, levels that are not already in v3.2.0, but added to v3.3.0. Using CATH terminology, the full data sets are denoted by SAll, whereas data sets *only* including sequences with less than 95% similarity are denoted by S95 (this does not apply to the unclassified set).

There are 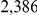 H-level families in v3.3.0, and 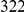 of these are singletons. Almost half of the families contain less than ten domains whereas a handful of families contain more than 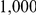 domains (the largest contains 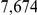 domains). Moreover, the distributions of family sizes are highly skewed and resemble power-laws (Fig. S10).

### 6. Classification using robust variables

The algorithm Random Forests [27] is used for classification. It is a probabilistic approach, that is, rerunning the algorithm might produce a (slightly) different result. A random forest builds 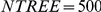 (user specified) classification trees based on a training set (where we always take the training set to be 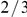 of the full data set) and uses majority voting for predicting the classes of the testing set.

Classification by random guessing is done by assigning family levels to domains according to family sizes, that is, the average success rate is the sum over 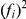, where 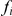 is the frequency of family 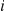.

### 7. Classification using flip sequences, amino acid sequences and Gauss integrals

For flip sequences, alignments were made using the Smith-Waterman algorithm with mismatch and gap penalties set to 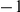 and match score to 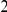. For amino acid sequences, BLOSUM40 was used. Let 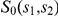 denote the score of the alignment of sequences 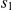 and 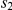. To facilitate a pairwise comparison of all sequences in CATH, regardless of lengths, we use the normalized score given by 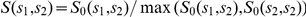, such that all scores are between 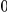 and 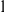.

For a given a domain, denote by 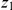 and 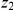 the normalized alignment scores of the domain to the nearest and second nearest H-levels in the v3.3.0 training set. A univariate measure was used to compare methods, 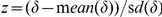, where 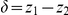 and the mean and sd are over all domains in the training set. For Gauss integrals, we used 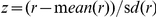 with 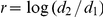, and 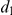 (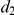) the distance to the (second) nearest H-levels [11]. Fig. S11 shows the distributions of the 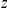-values for the three methods. Domains were classified according to their nearest H-level. A decision threshold was calibrated to achieve 95% sensitivity; all domains with 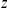 above the threshold are flagged as correctly identified if the nearest H-level corresponds to the true level. All domains with 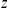 below the threshold are likewise correctly identifed as problematic if the nearest H-level is not the true level. Same procedure for C, A, and T-levels.

The three methods were combined into one method. Domains were classified according to the majority rule. If none of the methods agree, the method with the highest 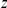-score decides the classification. A combined 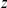-score was calculated as the maximum 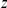-score of the agreeing methods. If all disagree, then the maximum 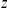-score is used. Fig. S10 shows the distribution of the combined 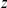-score.

## Supporting Information

Figure S1
**Each domain in the Pectate Lyase C-like topology was subjected to** 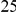 **independent modifications using the CONCOORD algorithm.** In the figure each column is a domain and the distribution of the normalized number of hydrogen in the modified structures is shown. The number is normalized relatively to the number observed in the original (unmodified) domain. The values corresponding to the eight outliers in Fig. 6 are highlighted in red, and all show a conspicuous decrease in the number of hydrogen bonds in the modified structures compared to the general trend of the remaining domains.(TIF)

Figure S2
**The deviation of** 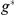 **and** 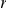 **from the observed values for eight randomly selected domains subjected to** 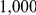 **modifications using the CONCOORD algorithm.** In general the modified values are centered around the observed values, though in some cases the distribution is biased to the left or right.(TIF)

Figure S3
**Distributions of the three quantities genus (** 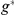 **), number of boundary components (** 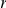 **), and number of twisted alpha carbon linkages (** 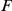 **) for all domains in v3.3.0.** Mainly beta and mixed alpha-beta have very similar distributions of 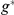 whereas mainly beta and mixed alpha-beta have very similar distributions of 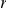.(TIF)

Figure S4
**Pairwise scatter plots of the five variables: the genus** 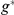 **, the number of boundary components** 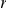 **, the number of twisted alpha carbon linkages** 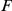 **, the number of residues** 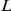 **and the Euler characteristic** 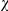 **for all domains in v3.3.0.** The variables 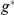 and 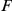 are positive or zero, 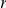 and 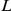 are strictly positive, and the Euler characteristic is at most 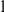. Further, the relationship 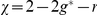 provides bounds, e.g. 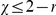. The plots indicate that the variables are capable of distinguishing CATH at the Class (C) level. For example, the 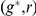, 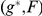, and 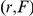 plots all show separation of the mainly alpha and the mainly beta classes with the mixed alpha-beta class falling somewhere between.(TIF)

Figure S5
**Wilcoxon plot corresponding to pairwise comparisons of the 1,161 H-levels comprising 10 or more domains with significance level** 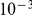 **(above diagonal) and** 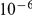 **(below diagonal).** Each row and column correspond to a H-level, and these are ordered by size in decreasing order. Colors indicate the number of variables (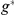, 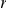, 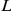, 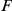) separating a pair of families at the given significance level: 0 (black), 1 (red), 2 (yellow), 3 (green), and 4 (white). Only every fifth H-level is used in the plot.(TIF)

Figure S6
**Plots of the variables** 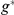 **,** 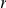 **, and** 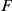 **, versus the number of alpha helices and beta sheets, respectively, for all domains in v3.3.0.** Separation of the mainly alpha and mainly beta classes with the mixed alpha-beta class falling somewhere between is observed. The higher genera observed in the mainly beta and mixed alpha-beta classes are mainly caused by beta sheets. Separation between classes is harder to spot on the plots with beta sheet counts.(TIF)

Figure S7
**Boxplots summarizing the success rates obtained on v3.3.0 using different subsets of variables for classification.** For all plots, an energy cut-off at 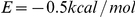 is used to determine hydrogen bonds. The last plot in the middle row is identical to Fig. 5. The last row shows success rates for 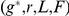 with alternative energy cut-offs used for determining hydrogen bonds.(TIF)

Figure S8 **Standard deviations of the genus and the number of boundary components for each H-level in v3.3.0 (SAll).** The standard deviations are generally not increasing with increasing H-level size, indicating that even large families are homogeneous. There is, however, more variation in the number of boundary components than in the genus.
(TIF)

Figure S9 **Correlation plots illustrating the difference between v3.2.0 (SAll) and the newly added domains in v3.3.0.** The left plot shows the sizes of the families in v3.2.0 test set versus the family sizes among the newly added domains and the right plot shows the corresponding performance rates. The new domains in v3.3.0 evidently have lower performance while family sizes roughly are proportional to those in v3.2.0.
(TIF)

Figure S10 **The distribution of H-level sizes in CATH 3.3.0 exhibits power-law behavior with many small levels and a few very large levels.**
(TIF)

Figure S11 **The distributions of normalized votes for all methods on the S95 training set.**
(TIF)

Table S1
**Comparison of all three classifiers on the non-redundant S95 subset of CATH (first three tables) as well as the entire CATH (SAll, last three tables).** At each level (C, A, T, and H) we split CATH v3.2.0 into two sets: For a level with N members, we used 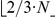 domains for training and the remaining N ï¿½ 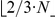 domains for testing. Note that for 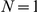 and 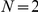, no domains are used for training. Therefore, in the CATH v3.2.0 test set as well as in the set of new domains in CATH v3.3.0, some domains do not have a classification present in the training set (despite the fact that the classification does exist in CATH v3.2.0). We call such domains unknown. All classifiers were trained to provide a 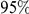 sensitivity on the training sets. For each set (S95 and SAll), the three tables show the following: **Top**: Performance, sensitivity, specificity, and unknown domains flagged as novel/problematic (in percent and in that order) on the CATH v3.2.0 training set. **Middle**: Similarly on the set of new domains in CATH v3.3.0 with classifications existing in CATH v3.2.0. **Bottom**: Some domains in CATH v3.3.0 have novel classifications not existing in CATH v3.2.0. This table summarized how many of these are flagged as novel/problematic by the three classifiers. Finally, the percentage of unclassified domains flagged by each method is shown. Note that there is only one set of unclassified domains, and this is used in both the S95 and the SAll case.(TIF)
