# Supplementary figures and images for: An Algebro-Topological Description of Protein Domain Structure
Source: PLoS One. 2011 May 24;6(5):e19670. doi: 10.1371/journal.pone.0019670 (PMC3101207; doi:10.1371/journal.pone.0019670)

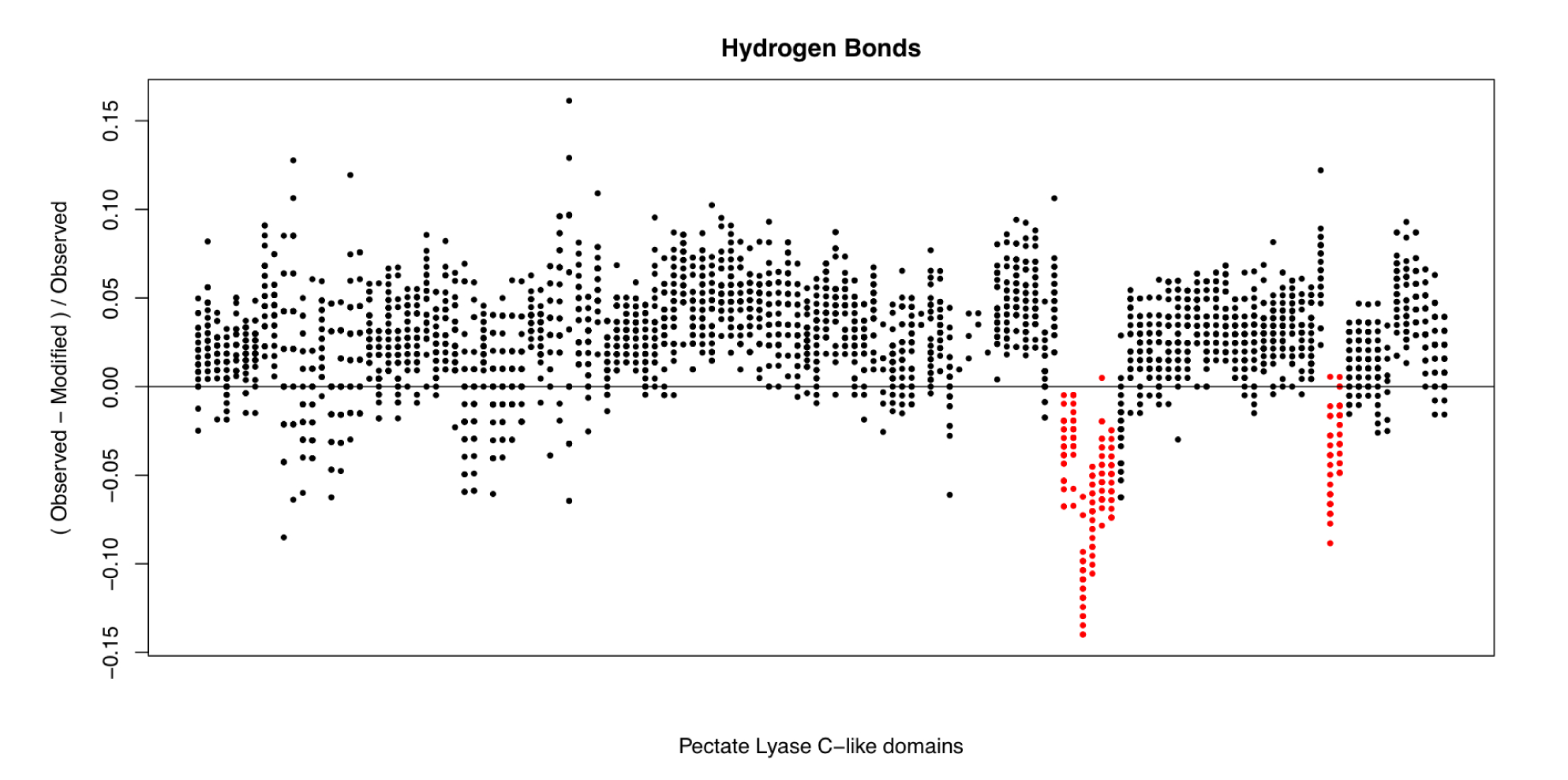

Supplement: Figure S1 — Each domain in the Pectate Lyase C-like topology was subjected to independent modifications using the CONCOORD algorithm. In the figure each column is a domain and the distribution of the normalized number of hydrogen in the modified structures is shown. The number is normalized relatively to the number observed in the original (unmodified) domain. The values corresponding to the eight outliers in Fig. 6 are highlighted in red, and all show a conspicuous decrease in the number of hydrogen bonds in the modified structures compared to the general trend of the remaining domains. (TIF) [file pone.0019670.s001.tif]

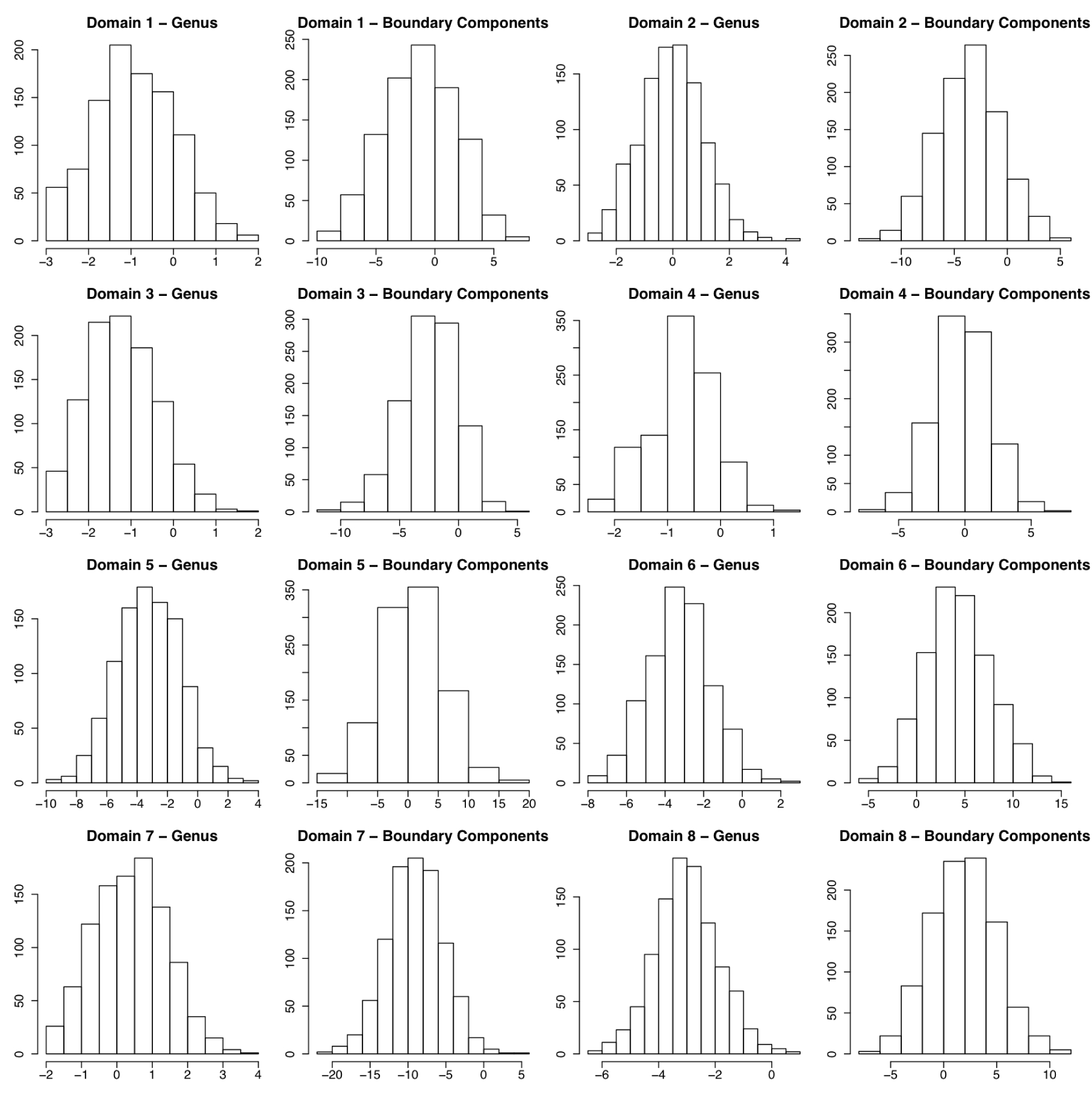

Supplement: Figure S2 — The deviation of and from the observed values for eight randomly selected domains subjected to modifications using the CONCOORD algorithm. In general the modified values are centered around the observed values, though in some cases the distribution is biased to the left or right. (TIF) [file pone.0019670.s002.tif]

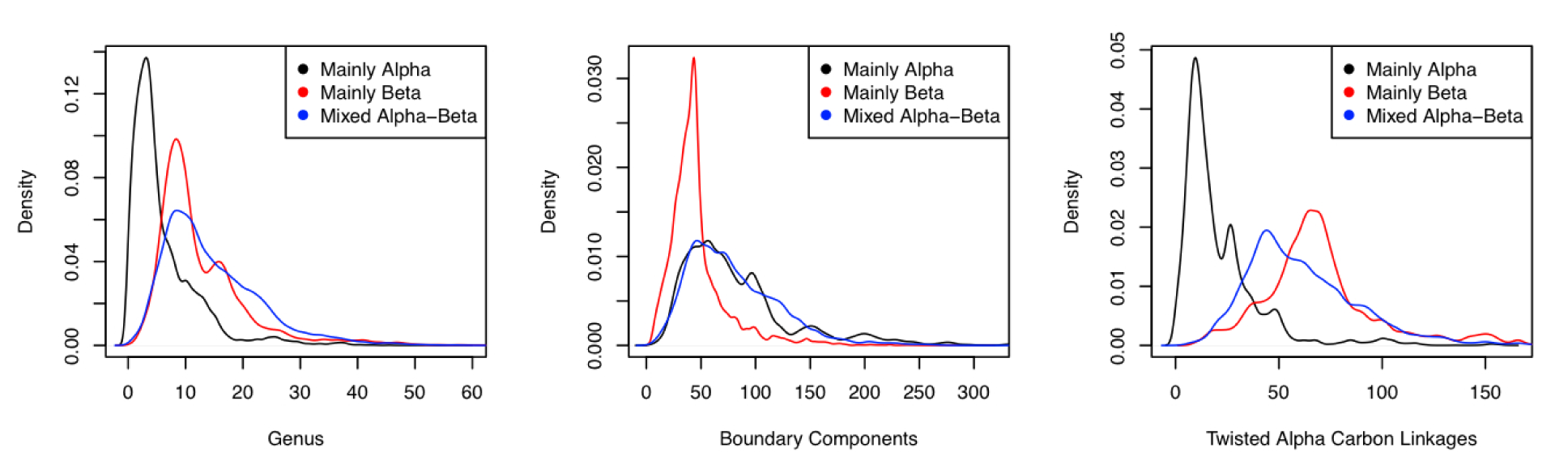

Supplement: Figure S3 — Distributions of the three quantities genus ( ), number of boundary components ( ), and number of twisted alpha carbon linkages ( ) for all domains in v3.3.0. Mainly beta and mixed alpha-beta have very similar distributions of whereas mainly beta and mixed alpha-beta have very similar distributions of . (TIF) [file pone.0019670.s003.tif]

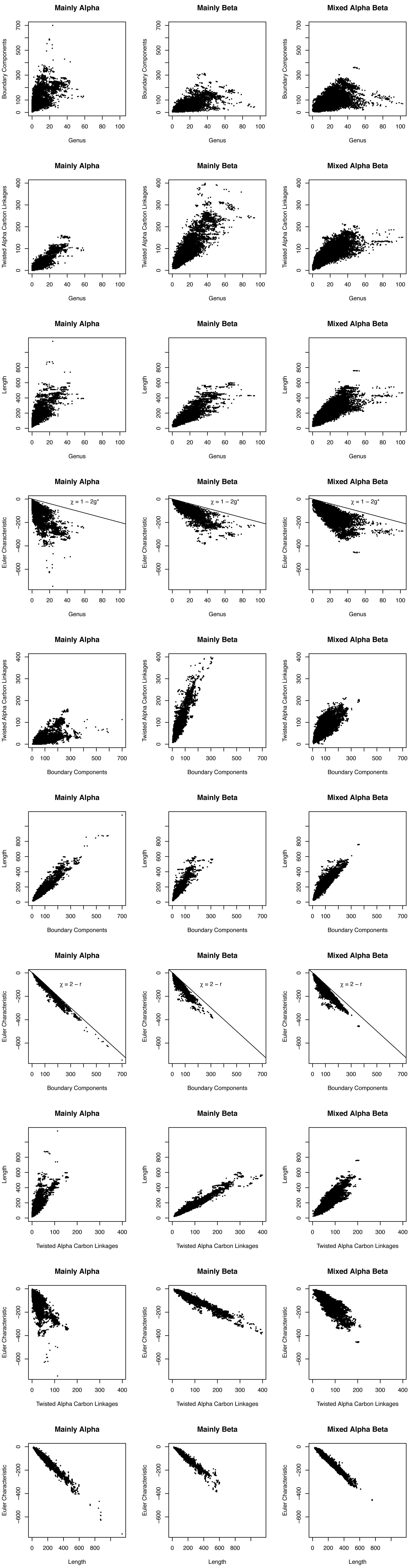

Supplement: Figure S4 — Pairwise scatter plots of the five variables: the genus , the number of boundary components , the number of twisted alpha carbon linkages , the number of residues and the Euler characteristic for all domains in v3.3.0. The variables and are positive or zero, and are strictly positive, and the Euler characteristic is at most . Further, the relationship provides bounds, e.g. . The plots indicate that the variables are capable of distinguishing CATH at the Class (C) level. For example, the , , and plots all show separation of the mainly alpha and the mainly beta classes with the mixed alpha-beta class falling somewhere between. (TIF) [file pone.0019670.s004.tif]

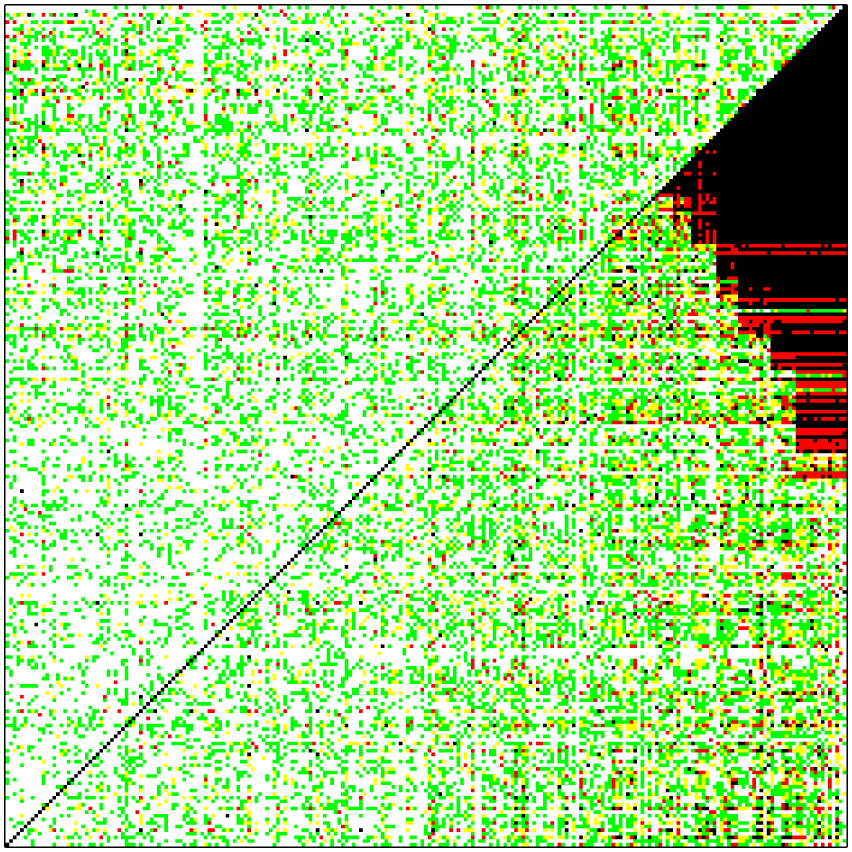

Supplement: Figure S5 — Wilcoxon plot corresponding to pairwise comparisons of the 1,161 H-levels comprising 10 or more domains with significance level (above diagonal) and (below diagonal). Each row and column correspond to a H-level, and these are ordered by size in decreasing order. Colors indicate the number of variables (, , , ) separating a pair of families at the given significance level: 0 (black), 1 (red), 2 (yellow), 3 (green), and 4 (white). Only every fifth H-level is used in the plot. (TIF) [file pone.0019670.s005.tif]

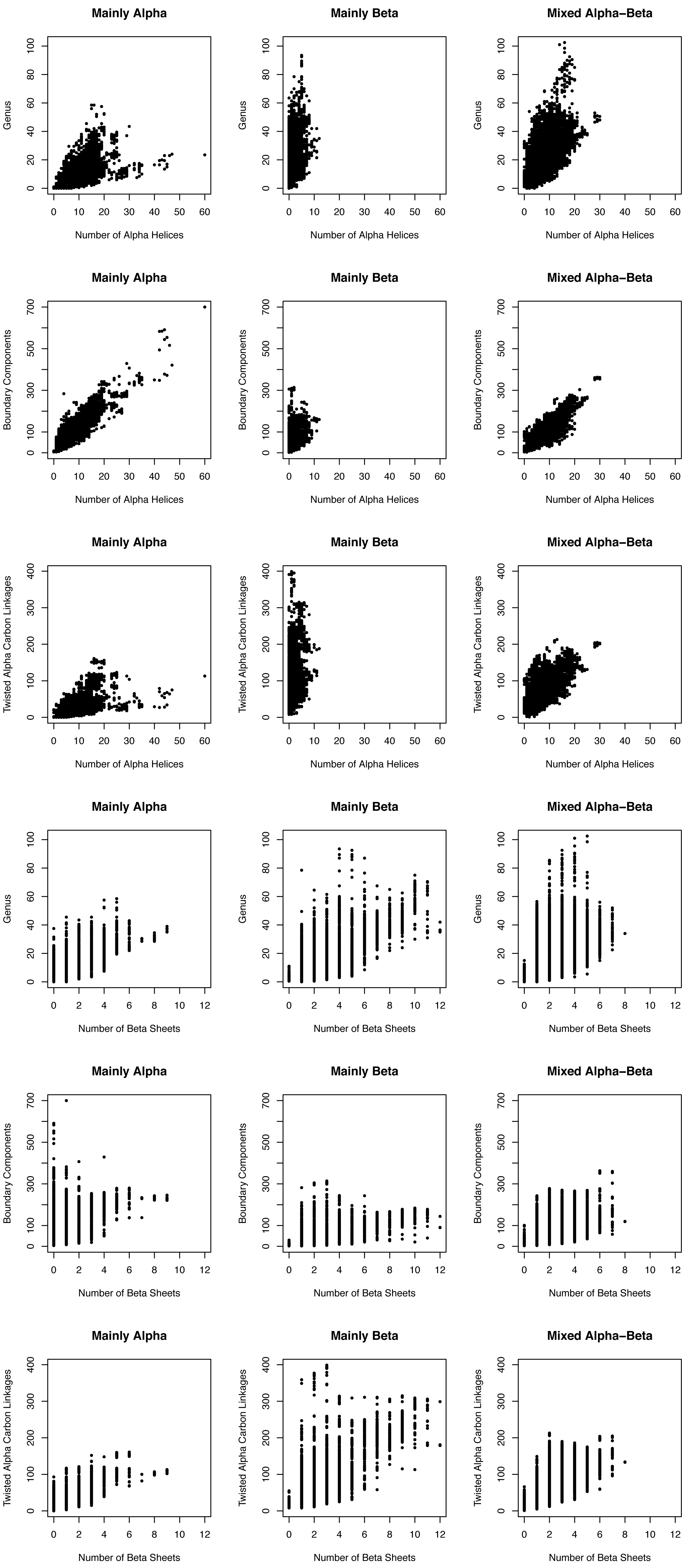

Supplement: Figure S6 — Plots of the variables , , and , versus the number of alpha helices and beta sheets, respectively, for all domains in v3.3.0. Separation of the mainly alpha and mainly beta classes with the mixed alpha-beta class falling somewhere between is observed. The higher genera observed in the mainly beta and mixed alpha-beta classes are mainly caused by beta sheets. Separation between classes is harder to spot on the plots with beta sheet counts. (TIF) [file pone.0019670.s006.tif]

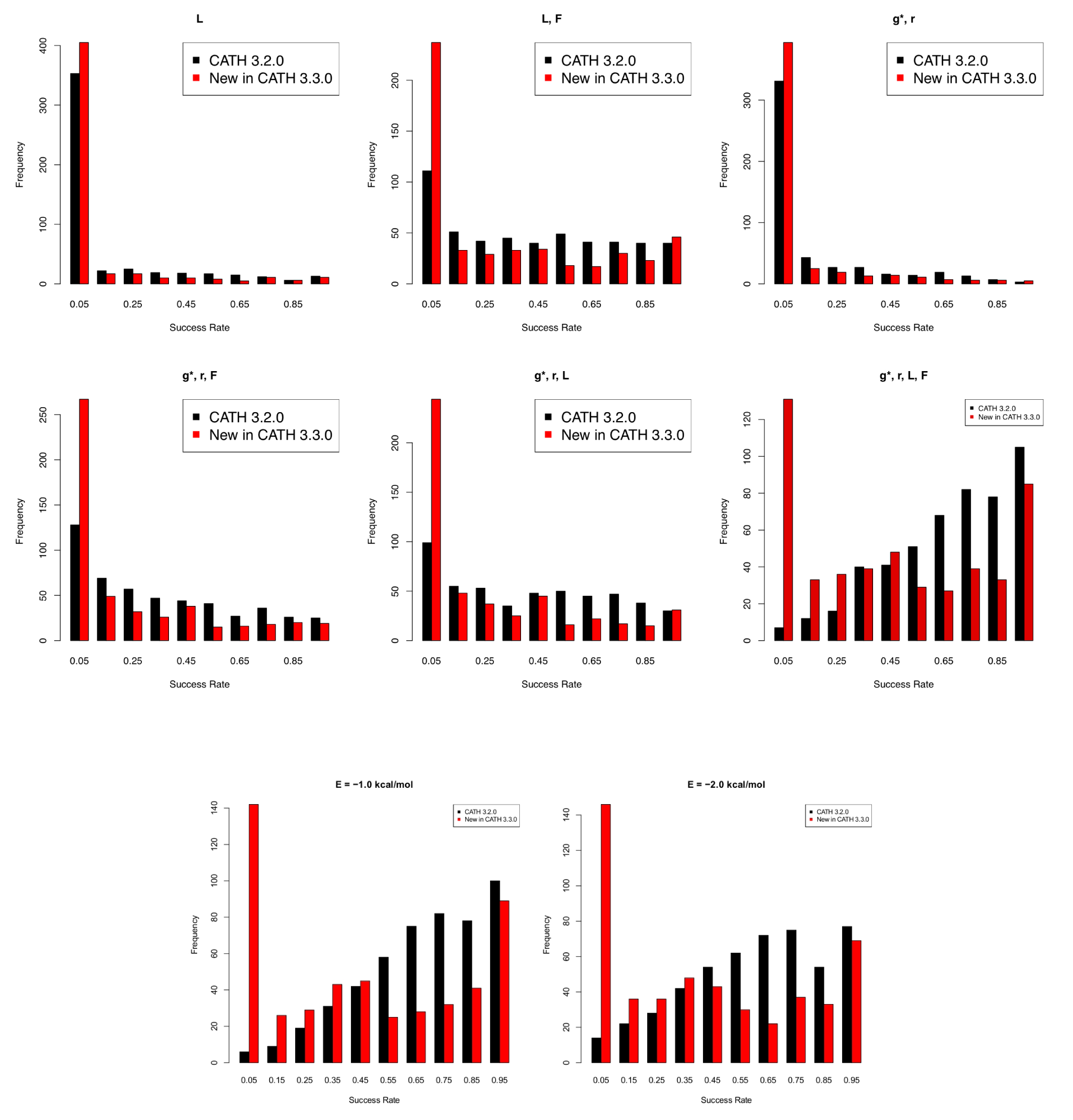

Supplement: Figure S7 — Boxplots summarizing the success rates obtained on v3.3.0 using different subsets of variables for classification. For all plots, an energy cut-off at is used to determine hydrogen bonds. The last plot in the middle row is identical to Fig. 5. The last row shows success rates for with alternative energy cut-offs used for determining hydrogen bonds. (TIF) [file pone.0019670.s007.tif]

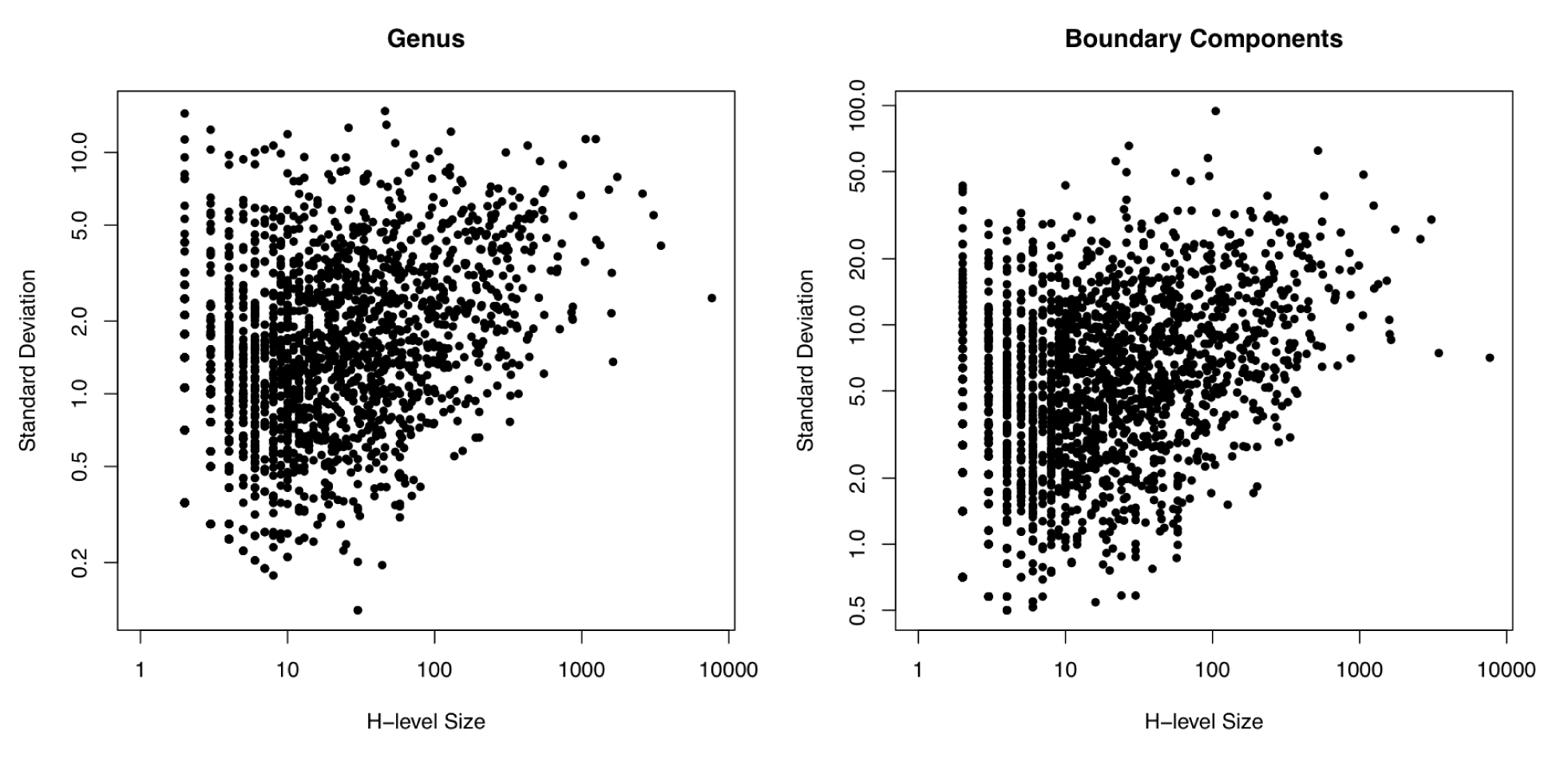

Supplement: Figure S8 — Standard deviations of the genus and the number of boundary components for each H-level in v3.3.0 (SAll). The standard deviations are generally not increasing with increasing H-level size, indicating that even large families are homogeneous. There is, however, more variation in the number of boundary components than in the genus. (TIF) [file pone.0019670.s008.tif]

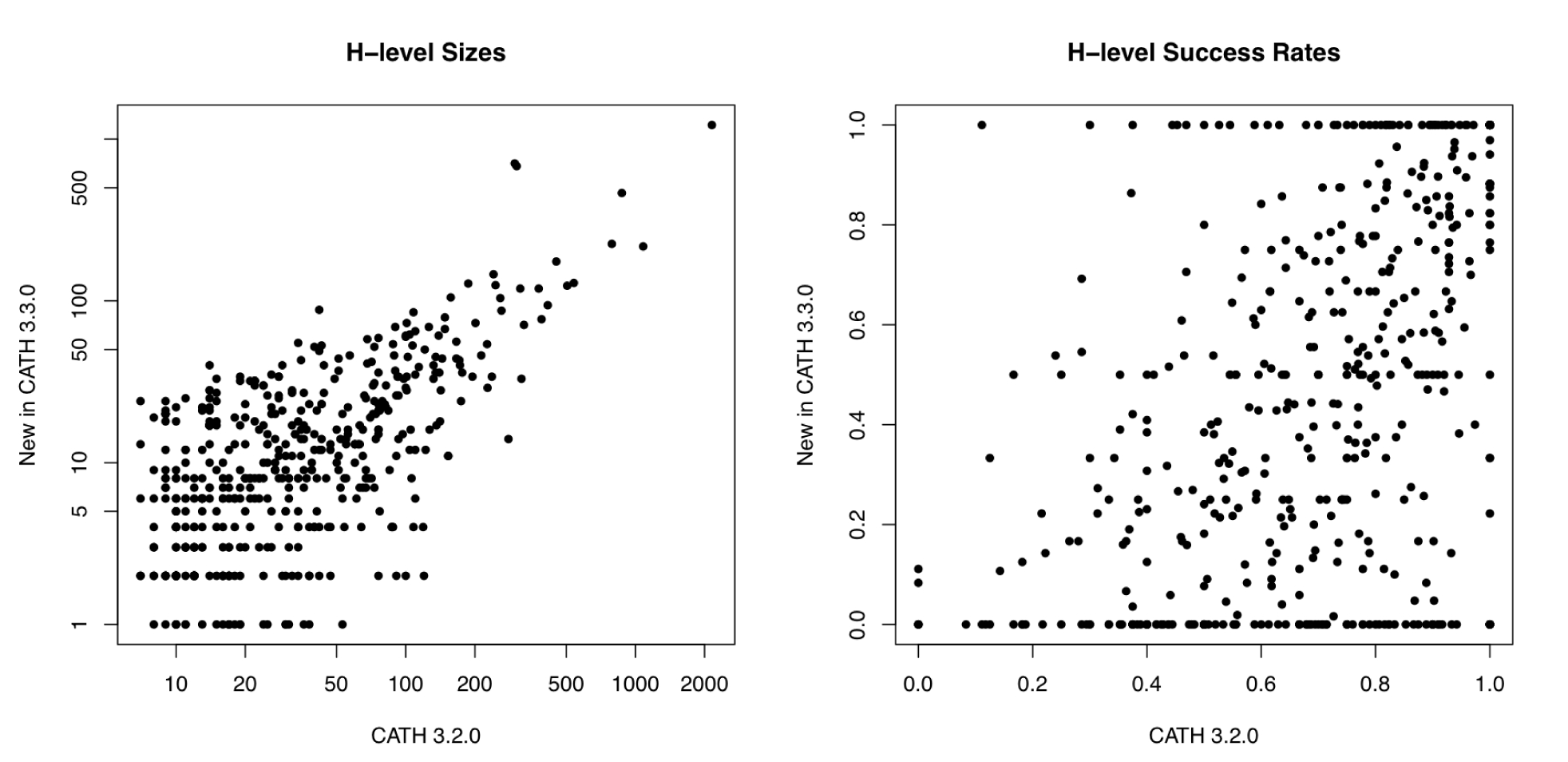

Supplement: Figure S9 — Correlation plots illustrating the difference between v3.2.0 (SAll) and the newly added domains in v3.3.0. The left plot shows the sizes of the families in v3.2.0 test set versus the family sizes among the newly added domains and the right plot shows the corresponding performance rates. The new domains in v3.3.0 evidently have lower performance while family sizes roughly are proportional to those in v3.2.0. (TIF) [file pone.0019670.s009.tif]

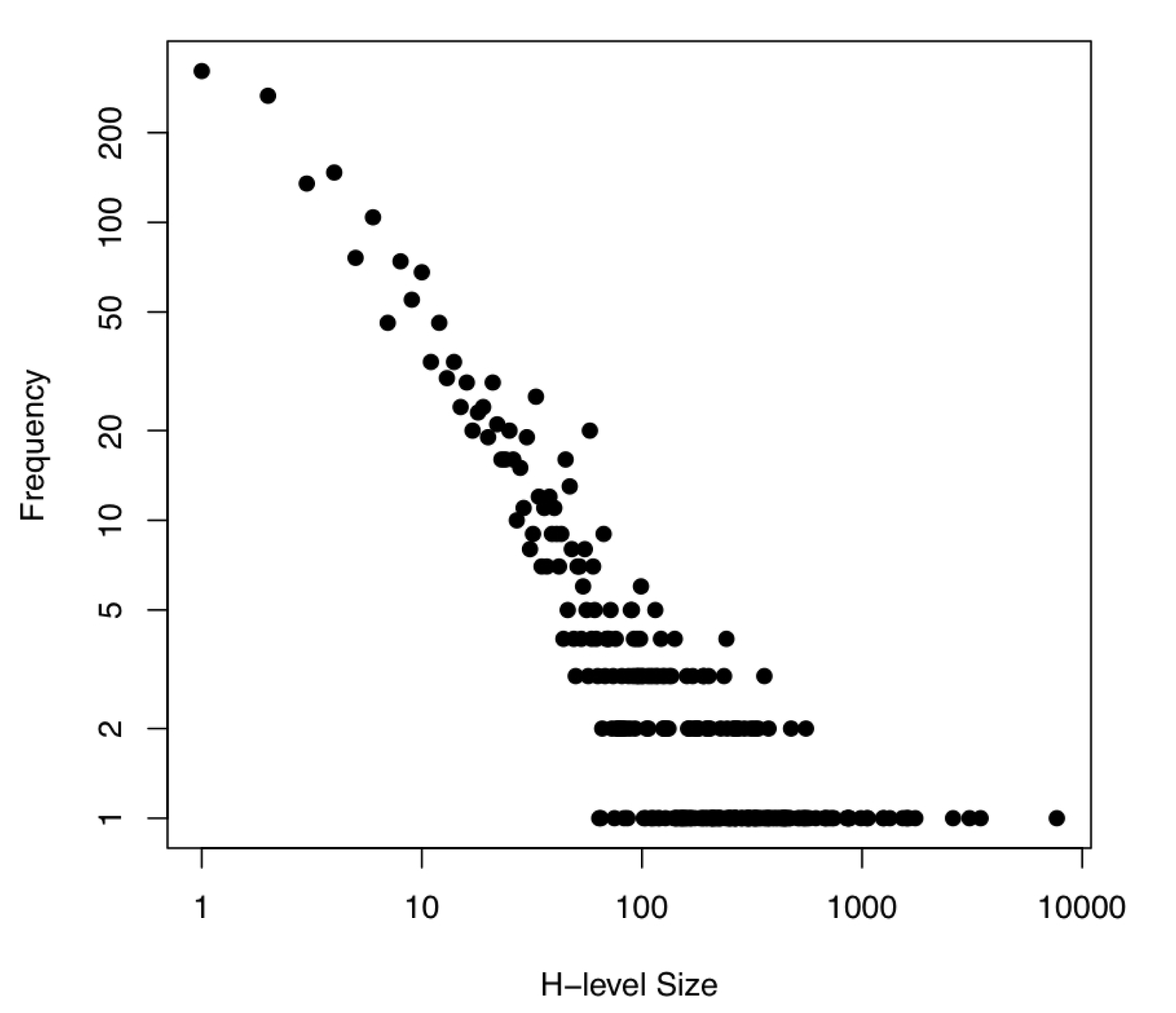

Supplement: Figure S10 — The distribution of H-level sizes in CATH 3.3.0 exhibits power-law behavior with many small levels and a few very large levels. (TIF) [file pone.0019670.s010.tif]

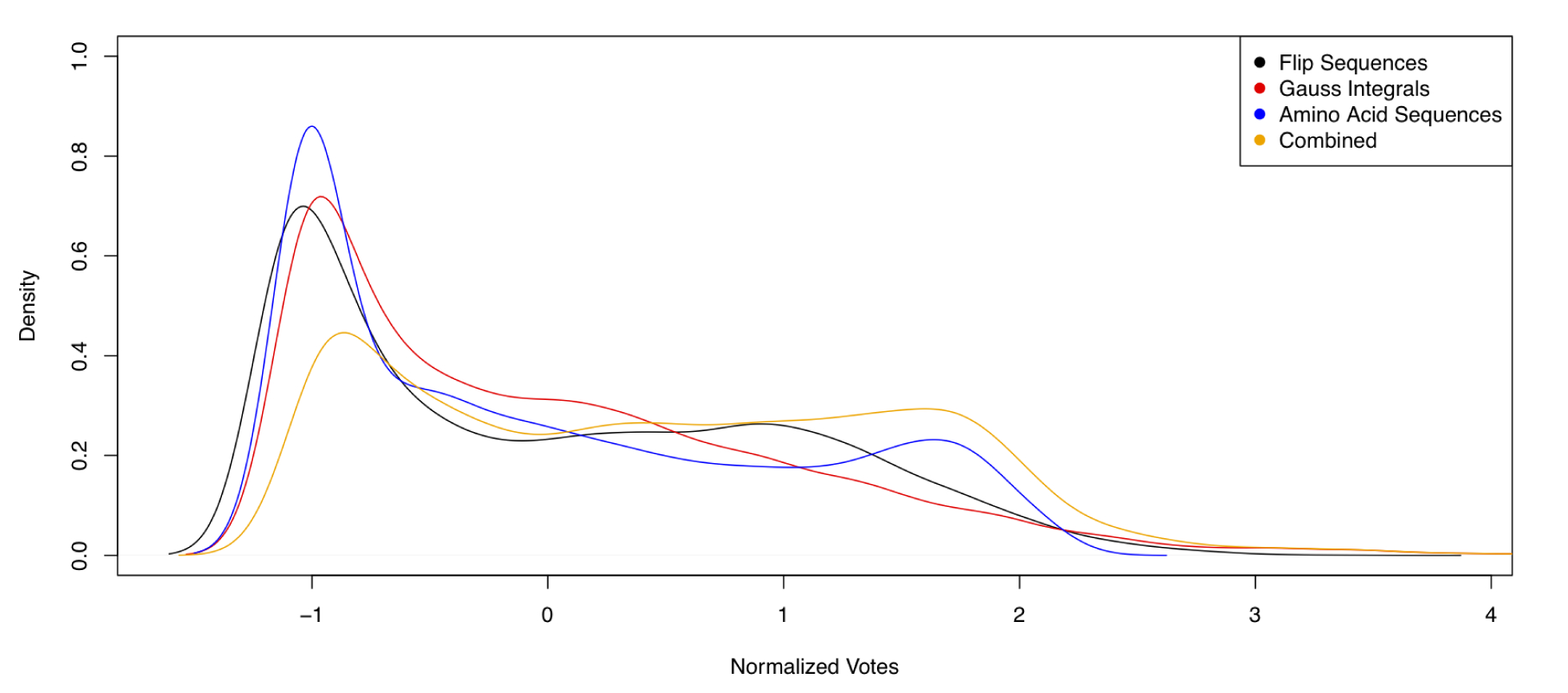

Supplement: Figure S11 — The distributions of normalized votes for all methods on the S95 training set. (TIF) [file pone.0019670.s011.tif]

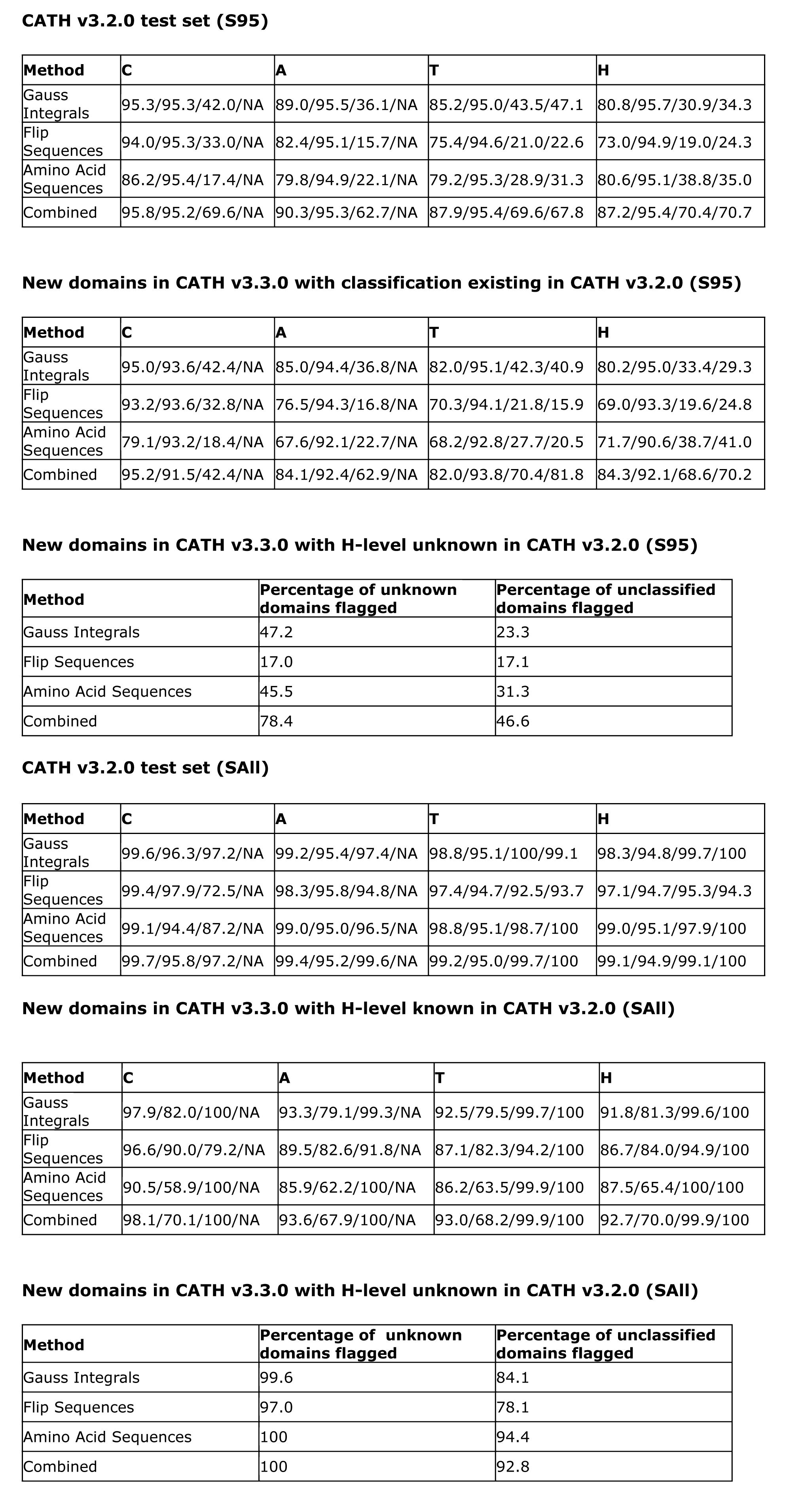

Supplement: Table S1 — Comparison of all three classifiers on the non-redundant S95 subset of CATH (first three tables) as well as the entire CATH (SAll, last three tables). At each level (C, A, T, and H) we split CATH v3.2.0 into two sets: For a level with N members, we used domains for training and the remaining N ï¿½ domains for testing. Note that for and , no domains are used for training. Therefore, in the CATH v3.2.0 test set as well as in the set of new domains in CATH v3.3.0, some domains do not have a classification present in the training set (despite the fact that the classification does exist in CATH v3.2.0). We call such domains unknown. All classifiers were trained to provide a sensitivity on the training sets. For each set (S95 and SAll), the three tables show the following: Top: Performance, sensitivity, specificity, and unknown domains flagged as novel/problematic (in percent and in that order) on the CATH v3.2.0 training set. Middle: Similarly on the set of new domains in CATH v3.3.0 with classifications existing in CATH v3.2.0. Bottom: Some domains in CATH v3.3.0 have novel classifications not existing in CATH v3.2.0. This table summarized how many of these are flagged as novel/problematic by the three classifiers. Finally, the percentage of unclassified domains flagged by each method is shown. Note that there is only one set of unclassified domains, and this is used in both the S95 and the SAll case. (TIF) [file pone.0019670.s012.tif]
